# A multi-agent equilibrium model in an incomplete market with discrete dividends: Applications to long-term discount curves

**DOI:** 10.1371/journal.pone.0343055

**Published:** 2026-07-24

**Authors:** Taiga Saito, Akihiko Takahashi

**Affiliations:** 1 Graduate School of Economics, Hitotsubashi University, Tokyo, Japan; 2 School of Interdisciplinary Mathematical Sciences / Graduate School of Advanced Mathematical Sciences, Meiji University, Tokyo, JapanCARF (Center for Advanced Research in Finance), The University of Tokyo, Tokyo, Japan; Yamanashi Gakuin University: Yamanashi Gakuin Daigaku, JAPAN

## Abstract

This paper develops a multi-agent equilibrium model in an incomplete market setting. The model incorporates dividend-paying securities whose dividend processes are interpreted as flows of consumption goods and can be driven by exogenously given factor processes. We consider an optimal consumption and portfolio problem for agents who have different views on fundamental risks and heterogeneous time preferences. Using a convex duality approach, we obtain expressions for the equilibrium state price density process, which subsequently yields the term structure of discount rates. To better reflect market practices, the model also incorporates discrete timing for dividend payments, consistent with semiannual or annual coupon schedules and policy decisions that typically occur at specific points during the year. As an application of the model, we provide numerical examples of long-term discount rates for valuing long-dated cashflows while exogenously incorporating the dynamics of factor processes that drive the dividend processes reflecting changes in the amount of government bonds available in the market. We examine how changes in the supply of government bonds affect the pricing of insurance products, including death benefits and pension annuities, through shifts in long-term discount rates. The numerical examples illustrate the qualitative implications of the model and are not intended to provide empirical findings.

## 1 Introduction

Yield curves for long-term maturities are important for discounting cashflows far into the future. However, long-term government bonds are generally illiquid, and the range of traded maturities is limited, so discount rates beyond the traded maturities must be assumed. For instance, Japanese government bonds are traded up to 40-year maturities, even though discount rates for longer maturities are often required when valuing long-term cashflows. In addition, yield curves for existing maturities are influenced by changes in government bond supply arising from central bank purchases, government issuance policies, and expectations regarding future supply operations. Therefore, a theoretical model that derives yield curve dynamics in response to changes in government bond supply is needed.

To address these issues, we propose a multi-agent equilibrium model to determine long-term interest rates for discounting future cashflows, incorporating the bond supply operations of governments and central banks. By exogenously incorporating dividend processes interpreted as flows of consumption goods, together with heterogeneous time preferences and differing views of representative institutional investors, we calculate the equilibrium interest rate, the market price of risk, and the resulting state price density process. This enables us to derive zero-coupon bond prices and construct the corresponding yield curve. As an application, we provide numerical examples illustrating the derivation of long-term discount rates for insurance pricing and how policy changes by the authorities affect insurance pricing through changes in long-term discount rates. Moreover, we incorporate discrete timing for dividend payments to reflect the practical schedule of coupon and dividend payments, such as semiannual or annual payments, and to capture policy decision timings that typically occur at specific points during the year. This feature makes the setting more realistic in reflecting the timing structure of actual financial markets.

For related literature on equilibrium models that derive the term structure of interest rates and address optimal consumption problems in complete or incomplete market settings, Vasicek [1], [2] developed a term structure model under complete market equilibrium with heterogeneous agents and a production process. Karatzas et al. [3] addressed the optimal consumption and portfolio problem for a single agent in an incomplete market using a convex dual approach, which incorporates fictitious securities.

Kizaki et al. [4] solved a complete market equilibrium with heterogeneous views and obtained the term structure of interest rates under differences in agents’ beliefs. Kizaki et al. [5] studied incomplete market equilibrium in a setting with utility from terminal wealth and one-time income, with applications to life-cycle investment and reinsurance pricing.

The financial modeling and methodological differences from Kizaki et al. [4], [5] are as follows. From a modeling perspective, this study examines the optimal consumption and investment problems of representative institutional investors, where dividend processes are specified so that their dynamics capture changes in the market supply of securities, allowing us to obtain the yield curve for discount rates under this setting. From a methodological perspective, the individual optimization problem is solved using a martingale approach for an incomplete market, and equilibrium is obtained by clearing the market for consumption goods. In particular, the equilibrium state price density process and the resulting yield curves are derived, reflecting agents’ different views on fundamental risks represented by Brownian motions and their heterogeneous time preferences.

As for empirical studies examining the effect of central banks’ outright purchases on the term structure of interest rates using reduced-form models, Nakano et al. [6] analyzed the impact of the Bank of Japan’s purchases of Japanese government bonds during the QQE period on the yield curve using a state-space model. Koeda and Sekine [7] evaluated a dynamic Nelson–Siegel model for Japanese government bond yields, suggesting that QQE negatively affects the time-dependent decay factor. Jarrow and Li [8] investigated the effect of quantitative easing on the U.S. term structure by estimating an arbitrage-free model that incorporates the price impacts of central bank bond purchases. Ray et al. [9] examined preferred habitat theory as a policy channel for quantitative easing, analyzing demand shocks within an equilibrium model. Joyce et al. [10] studied the Bank of England’s gilt purchases using an event-study approach combined with a portfolio balance model. In contrast to reduced-form approaches, Brummitt et al. [11] developed a structural model in which banks finance risky assets by issuing inside money. Their focus is on financial stability and equilibrium multiplicity rather than the term structure of interest rates, but their analysis highlights how central bank interventions can influence asset prices through balance-sheet channels.

Our study differs from previous research by deriving equilibrium long-term discount rates within an incomplete market model that incorporates agents’ heterogeneous time preferences and views on fundamental risks. Using this approach, we determine the long-term discount rate in market equilibrium when aggregate consumption equals aggregate dividends.

While we have presented numerical examples illustrating the model’s impact on insurance pricing through changes in long-term discount rates under specified parameter sets, conducting an empirical analysis based on this model remains an objective for future research.

The organization of this paper is as follows. Section 2 introduces the incomplete market model and addresses the individual optimal consumption and portfolio problem. Section 3 presents the derivation of interest rates and the market price of risk in equilibrium. Section 4 provides numerical examples illustrating how changes in the supply of government bonds affect insurance pricing through long-term discount rates. Section 5 concludes. The appendix contains the proofs of the propositions and theorems.

## 2 Discrete cash flow model

This section provides a discrete-time cashflow model, in which securities yield dividends at discrete points in time, and formulates the individual optimal consumption and portfolio problem of the agents. Specifically, Section 2.1 formulates the individual optimization problem of the agents under subjective belief in an incomplete market and Section 2.2 solves the optimal consumption and Section 2.3 provides optimal trading strategies and wealth processes.

Firstly, for a trading period [0,*T*], *T* > 0, let $\left(\Omega ,ℱ,\{{ℱ}_{t}{\}}_{0\leq t\leq T},P\right)$ be a filtered probability space satisfying the usual conditions. Let *W* be a Brownian motion of dimension *d*, where d≥N+1 and $\{{ℱ}_{t}{\}}_{0\leq t\leq T}$ be an augmented filtration generated by the Brownian motion. For j=1,…,N+1, let $r,\mu_{S}^{j},\sigma_{S}^{j}$ be ℛ-valued (${ℛ}^{1\times d}$-valued for $\sigma_{S}^{j}$) $\left\{{ℱ}_{t}\right\}$-progressively measurable processes and $\delta^{j}$ be a ℛ-valued nonnegative $\left\{{ℱ}_{t}\right\}$-progressively measurable process representing dividend processes paid at discrete timings $0<t_{1}<\ldots <t_{K}=T$ as follows.

Setting a cumulative dividend process $D^{j}$ as $D_{t}^{j}=\sum_{k:t_{k}\leq t}^{K}\delta_{t_{k}}^{j}$, and $dD_{t}^{j}:=D_{t}^{j}-D_{t-}^{j}$, we define the market value process of security *j*, $S^{j},\;j=1,\ldots ,N+1$, that yields dividends $\delta^{j}$, as a $\left\{{ℱ}_{t}\right\}$-progressively measurable processes satisfying an SDE


$$\begin{array}{c} dS_{t}^{j}=\mu_{S,t}^{j}S_{t}^{j}dt+S_{t}^{j}\sigma_{S,t}^{j}dW_{t}-dD_{t}^{j}\\ =r_{t}S_{t}^{j}dt+S_{t}^{j}\sigma_{S,t}^{j}(\theta_{t}dt+dW_{t})-dD_{t}^{j}, \end{array}$$ (1)

where θ is an ${ℛ}^{d}$-valued $\left\{{ℱ}_{t}\right\}$-progressively measurable process defined as


$$\begin{array}{c} \theta_{t}=\sigma_{S,t}^{⊤}(\sigma_{S,t}\sigma_{S,t}^{⊤}{)}^{-1}(\mu_{S,t}-r_{t})\in Range(\sigma_{S,t}^{⊤}). \end{array}$$ (2)

Here, $\sigma_{S,t}=(\sigma_{S,t}^{1⊤}\;\ldots \;\sigma_{S,t}^{N+1,⊤}{)}^{⊤}$, $\mu_{S,t}=(\mu_{1,t}\ldots \mu_{N+1,t}{)}^{⊤}$, and $Range(\sigma_{S,t}^{⊤})$ is a linear space spanned by $\sigma_{S,t}^{1}\;\ldots \;\sigma_{S,t}^{N+1}$. We also denote the price process of a money market account with instantaneous interest rate *r* by *B*, that is, $B_{t}=e^{\int_{0}^{t}r_{s}ds}$.

### 2.1 Individual optimization problem of the agents

Next, we formulate the individual optimization problems of the agents. We suppose that there are *I* (I≥2) agents who have log utility on *K* discrete-time consumption with heterogeneous views on the Brownian motion *W*.

Let $\pi^{i}$ and $\pi^{i,0}$ be ${ℛ}^{N+1}$ and ℛ valued $\left\{{ℱ}_{t}\right\}$ -progressively measurable processes satisfying $\int_{0}^{T}|\pi_{t}^{i}{|}^{2}ds$, $\int_{0}^{T}|\pi_{t}^{i,0}{|}^{2}ds,<\infty$, P−a.s., representing the position of the agent *i* on the *N* + 1 securities and the money market, respectively. Also, let $X^{i}$ be the wealth process of agent *i*, i.e., the total value of agent *i*’ portfolio. In detail, agent *i* invests $\pi^{i}$ of the wealth in the *N* + 1 securities and the rest $\pi^{i,0}$ in the money market account and continuously balances its position. That is,


$$\begin{array}{c} X_{t}^{i}=\pi_{t}^{i,0}+\pi_{t}^{i⊤}1, \end{array}$$

where 1 is an *N* + 1 dimensional column vector whose elements are 1.

In addition to the investments on *N* + 1 securities, at *K* discrete times $t_{1},\ldots ,t_{K}$, agent *i* consumes $c_{k}^{i}$ where $c_{k}^{i},\;k=1,\ldots ,K$ are ${ℱ}_{t_{k}}$- measurable nonnegative random variables satisfying $E[\sum_{k=1}^{K}(c_{k}^{i}{)}^{2}]<\infty$.

We consider the following admissible sets for the consumption $c_{k}^{i},\;k=1,\ldots ,K$ so that agent *i*’ wealth is always nonnegative, i.e.,


$$\begin{array}{c} X_{t}^{i}\geq 0,\;0\leq \forall t\leq T,\;P-a.s.. \end{array}$$ (3)

Namely, noting that the wealth process $X^{i}$ satisfies the following SDE,


$$\begin{array}{c} dX_{t}^{i}=\pi_{t}^{i,0}\frac{dB_{t}}{B_{t}}+\sum_{j=1}^{N+1}\pi_{t}^{i,j}\frac{dS_{t}^{j}+dD_{t}^{j}}{S_{t}^{j}}-dC_{t}^{i}\\ =r_{t}X_{t}^{i}dt+\pi_{t}^{i⊤}\sigma_{S,t}(\theta_{t}dt+dW_{t})-dC_{t}^{i},\;X_{0}^{i}=x_{0}^{i}>0, \end{array}$$ (4)

where


$$\begin{array}{c} dC_{t}^{i}=C_{t}^{i}-C_{t-}^{i};\;C_{t}^{i}=\sum_{k:t_{k}\leq t}c_{k}^{i}, \end{array}$$ (5)

we assume the following set of consumption processes $c^{i}$


$$\begin{array}{c} {𝒜}^{i}=\{c^{i}=\{c_{k}^{i}{\}}_{k=1,\ldots ,K}|E\left[\sum_{k=1}^{K}\frac{Z_{t_{k}}^{\theta^{i}}}{B_{t_{k}}}c_{k}^{i}\right]\leq x_{0}^{i},\\ \text{for all probability densities }Z^{\theta^{i}}\text{ for risk-neutral probability measures}\}, \end{array}$$ (6)

where the probability density $Z^{\theta^{i}}$ is of the form


$$\begin{array}{c} Z_{t}^{\theta^{i}}=e^{-\frac{1}{2}\int_{0}^{t}|\theta_{s}+\nu_{s}^{i}{|}^{2}ds-\int_{0}^{t}(\theta_{s}+\nu_{s}^{i})\cdot dW_{s}},\sigma_{S,t}\nu_{t}^{i}=0,\;\forall t\in [0,T], \end{array}$$

where $\nu^{i}$ is an ${ℛ}^{d}$-valued $\left\{{ℱ}_{t}\right\}$-progressively measurable process satisfying a weak version of Novikov’s condition (e.g., Corollary 3.5.14 in Karatzas and Shreve [12]) with θ and $\nu^{i}$ satisfying


$$\begin{array}{c} E\left[\int_{0}^{T}(|\theta_{s}{|}^{2}+|\nu_{s}^{i}{|}^{2})\eta_{s}^{i}ds\right]<\infty . \end{array}$$ (7)

**Remark 1.** *The above admissibility of the consumption process derives from the condition in* (3) *where the agent i does not go bankrupt, i.e., its wealth is always nonnegative. Since*


$$\begin{array}{c} X_{t}^{i}=x_{0}^{i}+\int_{0}^{t}\pi_{s}^{i,0}\frac{dB_{s}}{B_{s}}+\sum_{j=1}^{N+1}\int_{0}^{t}\pi_{s}^{i,j}\frac{dS_{s}^{j}+dD_{s}^{j}}{S_{s}^{j}}-\sum_{k:t_{k}<t}c_{k}^{i}\\ =x_{0}^{i}+\int_{0}^{t}r_{s}X_{s}^{i}ds+\int_{0}^{t}\pi_{s}^{i⊤}\sigma_{S,s}(\theta_{s}ds+dW_{s})-\sum_{k:t_{k}<t}c_{k}^{i}\geq 0, \end{array}$$

*multiplying both sides by the state price density* $H_{t}^{i}=\frac{Z_{t}^{\theta^{i}}}{B_{t}}$ *for t = T, applying Ito’s formula, and taking expectation yields*


$$\begin{array}{c} x_{0}^{i}\geq E\left[\sum_{k=1}^{K}c_{k}^{i}H_{t_{k}}^{i}\right]. \end{array}$$ (8)

*In detail, noting that*


$$\begin{array}{c} d(H_{t}^{i}X_{t}^{i})=X_{t}^{i}dH_{t}^{i}+H_{t}^{i}dX_{t}^{i}+d{\langle H^{i},X^{i}\rangle }_{t}\\ =X_{t}^{i}(-r_{t}H_{t}^{i}dt-H_{t}^{i}\theta_{t}^{⊤}dW_{t})\\ +H_{t}^{i}(r_{t}X_{t}^{i}dt+\pi_{t}^{i⊤}\sigma_{S,t}(\theta_{t}dt+dW_{t})-dC_{t}^{i})-H_{t}^{i}\pi_{t}^{i⊤}\sigma_{S,t}\theta_{t}dt\\ =H_{t}^{i}(-X_{t}^{i}\theta_{t}^{⊤}+\pi_{t}^{i⊤}\sigma_{S,t})dW_{t}-H_{t}^{i}dC_{t}^{i}. \end{array}$$

$$\begin{array}{c} E[X_{T}^{i}H_{T}]=x_{0}^{i}+E\left[\int_{0}^{T}H_{s}^{i}(-X_{s}^{i}\theta_{s}^{⊤}+\pi_{s}^{i⊤}\sigma_{S,s})dW_{s}\right]-E\left[\sum_{k=1}^{K}H_{t_{k}}^{i}c_{k}^{i}\right]\geq 0. \end{array}$$

*Therefore*


$$\begin{array}{c} x_{0}^{i}+E\left[\int_{0}^{T}H_{s}^{i}(-X_{s}^{i}\theta_{s}^{⊤}+\pi_{s}^{i⊤}\sigma_{S,s})dW_{s}\right] \end{array}$$ (9)

*Since* $\int_{0}^{t}H_{s}^{i}(-X_{s}^{i}\theta_{s}^{⊤}+\pi_{s}^{i⊤}\sigma_{S,s})dW_{s}$ *is a local martingale bounded from below, thus a supermartingale,*


$$\begin{array}{c} E\left[\int_{0}^{T}H_{s}^{i}(-X_{s}^{i}\theta_{s}^{⊤}+\pi_{s}^{i⊤}\sigma_{S,s})dW_{s}\right]\leq 0, \end{array}$$

we have


$$\begin{array}{c} x_{0}^{i}\geq E\left[\sum_{k=1}^{K}H_{t_{k}}c_{k}^{i}\right]. \end{array}$$

**Remark 2.** *In this study, we discretely set the consumption timing to align with the timing of the dividends paid, where the dividends are assumed to be paid as consumption goods. In particular, we consider the clearing of consumption goods at each discrete time, which indicates that the total dividend amount is consumed by the agents at each time. The matched timing between consumption and dividends and the clearing condition make it possible to obtain the state price density process in equilibrium.*

*For dividend processes, we model them as discrete-time processes to represent the feature of periodically paid dividends. The dividend reflects supply change of the securities such as outstanding notional changes by purchase of government bonds by the central bank and redemption, and new issuance of the bonds by the government at planned timings.*

For the utility function, we assume that each agent *i* has the following log utility u(x)=logx on consumption with subjective belief $\lambda^{i}$ on the Brownian motion *W* with heterogeneous time preferences $\alpha^{i}$.

In detail, the agent maximizes the sum of its expected utility on the discrete consumption with discounting for time preference $\alpha_{t}^{i}=e^{-\beta_{i}t}$, where $\beta_{i}>0$. The probability density $\eta_{T}^{i}$ for subjective belief $\lambda^{i}$ of agent *i*, is defined as


$$\begin{array}{c} \eta_{T}^{i}=\exp\left(\int_{0}^{T}\lambda_{s}^{i}\cdot dW_{t}-\frac{1}{2}\int_{0}^{T}|\lambda_{s}^{i}{|}^{2}ds\right), \end{array}$$

where $\lambda^{i}$ is an ${ℛ}^{d}$-valued $\left\{{ℱ}_{t}\right\}$-progressively measurable process satisfying a weak version of Novikov’s condition (see, e.g., Corollary 3.5.14 in Karatzas and Shreve [12]). Here, $\lambda^{i}$ represents the subjective views of the agent *i* on the Brownian motion *W*. Namely, for the probability measure $P^{i}$ defined as


$$\begin{array}{c} \frac{dP^{i}}{dP}=\eta_{T}^{i}, \end{array}$$ (10)

by Girsanov’s theorem, $W^{P^{i}}$ defined as $dW_{t}=dW_{t}^{P^{i}}+\lambda^{i}dt$ is a $P^{i}$-Brownian motion and $\lambda^{i}$ indicates agent *i*’ bias on the Brownian motion *W*.

For related studies dealing with subjective views by change of probability measure and their estimation, Nakatani et al. [13] estimated the market sentiments represented by a way of Girsanov’s measure transformation in the Japanese government bond market.

Also, Kizaki et al. [4] further dealt with subjective views in the form of Girsanov’s transformation of the probability measure as the inf-sup and sup-sup problem, where the conservative view is expressed as taking infimum on the objective function with respect to the change of the probability measure, and the aggressive view is expressed as taking supremum instead. For example, in Kizaki et al. [4], $\inf_{\lambda^{i}}\sup_{\pi }E^{P^{i}}[u(X_{T}^{\pi })]$ is considered for the conservative sentiment of the agent, while $\sup_{\lambda^{i}}\sup_{\pi }E^{P^{i}}[u(X_{T}^{\pi })]$ is considered for the aggressive sentiment.

The alternative approach is a robust control for portfolio optimization (e.g., Hansen and Sargent [14]) in which the belief is considered as a model uncertainty and the minimum is taken to be conservative about the model risk.

Then, we describe individual optimization problem of the agent *i* as follows.

**Individual optimization problem**

Maximize


$$\begin{array}{c} \sum_{k=1}^{K}E[\eta_{t_{k}}^{i}\alpha_{t_{k}}^{i}\log c_{k}^{i}], \end{array}$$ (11)

with respect to $c^{i}\in {𝒜}^{i}$, where ${𝒜}^{i}$ is defined in (6).

This individual optimization problem indicates that the agent *i* aims to maximize its expected utility in its consumption at discrete times with the time preference $\beta^{i}$ by choosing the consumption amount while continuously trading *N* + 1 securities and the money market account.

**Remark 3.** *Although we may adopt exponential utility or power utility, in this study, we adopt log utility for the following reasons. Firstly, in individual optimization, because the problem is in an incomplete market setting, we take infimum concerning the possible state price density processes* $H^{i}$ *parametrized with* $\nu^{i}$ *orthogonal to the volatility vectors of the underlying asset price processes, while taking supremum on the consumption and trading strategies on expected utility. This infimum part of the individual optimization in an incomplete market setting is solved in the case of the log-utility.*

*While it remains unsolved in the power utility and exponential utility cases, in the log-utility case, thanks to the explicit expression of the consumption process for the individual agent after solving the individual optimization problem in an incomplete market setting.*

*Moreover, by incorporating the views on fundamental risks for the agents in the log utility case, we can obtain the equilibrium state price density process that reflects the agents’ views on the Brownian motion.*

### 2.2 Optimal consumption for the individual optimization

This section provides an optimal consumption process for this individual optimization problem (11). In the following, we consider the following primal problem for the individual optimization.


$$\begin{array}{c} \sup_{c^{i}}\inf_{y^{i}>0,\nu^{i},\sigma_{S}\nu^{i}=0}\sum_{k=1}^{K}E[\eta_{t_{k}}^{i}\alpha_{t_{k}}^{i}\log c_{k}^{i}]+y^{i}\left(x_{0}^{i}-E[\sum_{k=1}^{K}H_{t_{k}}^{i}c_{k}^{i}]\right), \end{array}$$ (12)

To solve this primal problem, we first solve the dual problem and later confirm that the obtained solution is optimal.

**Remark 4.** *The primal optimization problem describes the individual optimization problem* (11) *for the following reasons. If the given* $c^{i}$ *does not satisfy the budget constraint in* (6) *for some* $H^{i}$*, the inf part is* −∞ *by taking* $y^{i}$ *any large value. Thus, the sup part indicates that the sup is taken with respect to* $c^{i}$ *in* ${𝒜}^{i}$ *in* (6). *Hence, the primal problem expresses the individual optimization problem* (11) *where taking supremum on the expected utility with respect to* $c^{i}$ *satisfying the budget constraint in* (6). The dual problem is described and solved as follows.

**Dual problem**


$$\begin{array}{c} \inf_{y^{i}>0,\nu^{i},\sigma_{S}\nu^{i}=0}\sup_{c^{i}}\sum_{k=1}^{K}E[\eta_{t_{k}}^{i}\alpha_{t_{k}}^{i}\log c_{k}^{i}]+y^{i}\left(x_{0}^{i}-E[\sum_{k=1}^{K}H_{t_{k}}^{i}c_{k}^{i}]\right), \end{array}$$ (13)

**Proposition 1.** $c^{i,*}$, $\nu^{i}$, $y^{i}$ *set as*


$$\begin{array}{c} c_{k}^{i,*}=\frac{x_{0}^{i}}{\sum_{k=1}^{K}\alpha_{t_{k}}^{i}}\frac{\alpha_{t_{k}}^{i}Z_{t_{k}}^{i}B_{t_{k}}}{Z_{t_{k}}^{\theta }}>0,\\ \nu_{t}^{i}=-\overset{i,⟂}{\underset{t}{\hat{\lambda }}},\\ y^{i}=\frac{\sum_{k=1}^{K}\alpha_{t_{k}}^{i}}{x_{0}^{i}}, \end{array}$$

*attain the inf-sup dual problem* (13)*, where*


$$\begin{array}{c} Z_{t}^{i}=\exp(-\frac{1}{2}\int_{0}^{t}|\overset{i}{\underset{s}{\hat{\lambda }}}{|}^{2}ds+\int_{0}^{t}\overset{i}{\underset{s}{\hat{\lambda }}}\cdot dW_{s}), \end{array}$$ (14)

$$\begin{array}{c} Z_{t}^{\theta }=\exp(-\frac{1}{2}\int_{0}^{t}|\theta_{s}{|}^{2}ds-\int_{0}^{t}\theta_{s}\cdot dW_{s}). \end{array}$$ (15)

*Here,* $\overset{i,⟂}{\underset{t}{\hat{\lambda }}}$ *is an orthogonal part of* $\lambda_{t}^{i}$ *to the linear space spanned by* $\sigma_{S,t}$*, i.e.,* $\lambda_{t}^{i}=\overset{i}{\underset{t}{\hat{\lambda }}}⊕\overset{i,⟂}{\underset{t}{\hat{\lambda }}}$, $\overset{i}{\underset{t}{\hat{\lambda }}}\in Range(\sigma_{S,t}^{⊤}),\sigma_{S,t}\overset{i,⟂}{\underset{t}{\hat{\lambda }}}=0$.

**Proof**. See Appendix A.1. □

Then, by a convex duality approach, we confirm that the solution obtained $c^{i,*}$, $\nu^{i}$, and $y^{i}$, of the dual problem (13) is also a solution of the primal problem (12) as follows.

**Theorem 1.** *The solution of the dual problem* (13)*,* $c^{i,*}$, $\nu^{i}$*,* $y^{i}$ *obtained as*


$$\begin{array}{c} c_{k}^{i,*}=\frac{x_{0}^{i}}{\sum_{k=1}^{K}\alpha_{t_{k}}^{i}}\frac{\alpha_{t_{k}}^{i}Z_{t_{k}}^{i}B_{t_{k}}}{Z_{t_{k}}^{\theta }}>0, \end{array}$$ (16)

$$\begin{array}{c} \nu_{t}^{i}=-\overset{i,⟂}{\underset{t}{\hat{\lambda }}},\;0\leq t\leq T\\ y^{i}=\frac{\sum_{k=1}^{K}\alpha_{t_{k}}^{i}}{x_{0}^{i}}, \end{array}$$

*also attains the sup-inf of the primal problem* (12).

**Proof**. See Appendix A.2. □

### 2.3 Optimal wealth and portfolio processes of the agents

Finally, for the optimal consumption $c^{i,*}$ obtained as a solution to the primal problem, the corresponding portfolio process $\left(\pi^{i,*},\pi^{i,0,*}\right)$ along with the optimal wealth, which is nonnegative, is obtained as follows.

**Theorem 2.** *Under the assumption that* $rank(\sigma_{S,t})=N+1$ *for* 0≤t≤T*, the optimal wealth process* $X^{i,*}$ *and the portfolio process* $\left(\pi^{i,*},\pi^{i,0,*}\right)$ *corresponding to the optimal consumption* $c^{i,*}$ *in* (16)*, i.e.,* $\left(\pi^{i,*},\pi^{i,0,*}\right)$ *that generates wealth* $X^{i,*}$ *satisfying the condition of the nonnegative wealth process in* (3)*, for the individual optimization problem* (11) *are given as follows.*


$$\begin{array}{c} X_{t}^{i,*}=\frac{B_{t}Z_{t}^{i}}{Z_{t}^{\theta }}\;\frac{x_{0}^{i}}{\sum_{k=1}^{K}\alpha_{t_{k}}^{i}}\sum_{k:t_{k}\geq t}^{K}\alpha_{t_{k}}^{i}>0, \end{array}$$ (17)

$$\begin{array}{c} \pi_{t}^{i,*}=X_{t}^{i,*}(\sigma_{S,t}\sigma_{S,t}^{⊤}{)}^{-1}\sigma_{S,t}(\overset{i}{\underset{t}{\hat{\lambda }}}+\theta_{t}), \end{array}$$ (18)

*and*


$$\begin{array}{c} \pi_{t}^{i,0,*}=X_{t}^{i,*}-\pi_{t}^{i,*⊤}1. \end{array}$$ (19)

**Proof**. See Appendix A.3.□

**Remark 5.** *First,* $X_{t}^{i,*}$ *in* (17) *corresponding to* $\left(c^{i,*},\pi^{i,*},\pi^{i,0,*}\right)$ *in* (16)*,* (18) *and* (19) *satisfies* $X_{t}^{i,*}>0,\;0\leq \forall t\leq T,\;P-a.s.$ *Since* $c^{i,*}$ *is the optimal consumption in the admissible set* ${𝒜}_{i}$*, in which all the consumption processes in* (4)*,* (5) *with nonnegative condition* (3) *are included as shown in Remark 1, it follows that* $\left(c^{*},\pi^{i,*},\pi^{i,0,*}\right)$ *is the optimal consumption and portfolio among triplets* $\left(c,\pi^{i},\pi^{i,0}\right)$ *for a problem* $\sup_{\left(c^{i},\pi^{i},\pi^{i,0}\right)}\sum_{k=1}^{K}E[\eta_{t_{k}}^{i}\alpha_{t_{k}}^{i}\log c_{k}^{i}]$ *subject to* (4)*,* (5)*, and the nonnegative wealth condition* (3)*. In other words, we solve the individual optimization of the candidates in the admissible set* ${𝒜}^{i}$*, which is a larger set that includes the consumption and portfolio processes with which the wealth process never goes bankrupt. Thus, the optimal solution is found in the broader set of consumption and portfolio processes. Then we confirm that with the optimal solution found in the broader set of consumption and portfolio processes, the wealth process is positive, that is,* $X_{t}^{i,*}>0,\;0\leq t\leq T$ *based on the expression of the optimal wealth process.*

*Thus, the obtained consumption and portfolio processes are optimal among the initial intended set, i.e., the strategies with which the wealth process is always nonnegative.*

## 3 Market clearing condition and equilibrium

This section provides expressions of the state-price density process, which derives the interest rate and the market price of risk, and yield curves corresponding to zero-coupon bond prices in equilibrium. Section 3.1 obtains the state-price density process in equilibrium by imposing the market clearing condition on the optimal consumption processes of agents $c^{i,*},\;i=1,\ldots ,I$ in Theorem 1 and derives interest rate, zero coupon bond prices, and the corresponding yield curves in equilibrium. Section 3.2 presents market equilibrium between the market values of the securities and the aggregate wealth of the agents. Section 3.3 investigates the recursive structure of the volatility processes of the securities in equilibrium.

Specifically, we consider the following market clearing condition in which aggregate consumption over agents is equal to dividends from the securities.

**Market clearing condition**

At each discrete time $t_{k},\;k=1,\ldots ,K$, the following equation holds.


$$\begin{array}{c} \sum_{i=1}^{I}c_{k}^{i,*}=\sum_{j=1}^{N+1}\delta_{t_{k}}^{j}\equiv \delta_{t_{k}}. \end{array}$$ (20)

When the optimal consumption processes of the agents satisfy this clearing condition, we call the market in equilibrium.

(20) indicates that the consumption goods, which are paid as dividends, are consumed by the representative shareholders. In the model, representative shareholders consume the goods to maximize their utility while they receive dividends from their holding securities. Thus, the market clearing condition supposes that the aggregate dividends are consumed by the agents at discrete times.

### 3.1 State-price density process and yield curves in equilibrium

First, we obtain the following expressions for the interest rate *r* and the density process $Z^{\theta }$, which represents the common part of the risk-neutral probability measure and incorporates the market price of risk θ in equilibrium.

**Theorem 3.** *In equilibrium,* $Z^{\theta }$ *in* (15) *is expressed as*


$$\begin{array}{c} Z_{t_{k}}^{\theta }=\frac{B_{t_{k}}}{\delta_{t_{k}}}\sum_{i=1}^{I}\frac{x_{0}^{i}\alpha_{t_{k}}^{i}Z_{t_{k}}^{i}}{\sum_{k=1}^{K}\alpha_{t_{k}}^{i}} \end{array}$$

*for* k=1,…,K *and for* $t\in (t_{k},t_{k+1}]$,


$$\begin{array}{c} Z_{t}^{\theta }=E\left[\frac{B_{t_{k}}}{\delta_{t_{k}}}\sum_{i=1}^{I}\frac{x_{0}^{i}\alpha_{t_{k}}^{i}Z_{t_{k}}^{i}}{\sum_{k=1}^{K}\alpha_{t_{k}}^{i}}|{ℱ}_{t}\right]. \end{array}$$ (21)

*Also the associated state price density process H is given by*


$$\begin{array}{c} H_{t}=E\left[\frac{1}{\delta_{t_{k}}}\sum_{i=1}^{I}\frac{x_{0}^{i}\alpha_{t_{k}}^{i}Z_{t_{k}}^{i}}{\sum_{k=1}^{K}\alpha_{t_{k}}^{i}}|{ℱ}_{t}\right]. \end{array}$$ (22)

*Moreover, supposing that the interest rate process r is piece-wise ${ℱ}_{t_{k-1}}$-measurable random variable between the discrete times $\left(t_{k-1},t_{k}\right]$, r is expressed as*


$$\begin{array}{c} r_{t}=\frac{1}{\left(t_{k}-t_{k-1}\right)}\log\left[\frac{\frac{1}{\delta_{t_{k-1}}}\sum_{i=1}^{I}\frac{x_{0}^{i}\alpha_{t_{k-1}}^{i}Z_{t_{k-1}}^{i}}{\sum_{k=1}^{K}\alpha_{t_{k}}^{i}}}{E\left[\frac{1}{\delta_{t_{k}}}\sum_{i=1}^{I}\frac{x_{0}^{i}\alpha_{t_{k}}^{i}Z_{t_{k}}^{i}}{\sum_{k=1}^{K}\alpha_{t_{k}}^{i}}|{ℱ}_{t_{k-1}}\right]}\right]\text{for }t\in (t_{k-1},t_{k}]. \end{array}$$ (23)

By expression of the state price density process (22), the price of the zero coupon bond P(0,𝒯) and the yield 𝒴(0,𝒯) for maturity 𝒯 at *t* = 0 are described as follows.


$$\begin{array}{cc} P(0,𝒯) & =E\left[H_{𝒯}\right]=E\left[\frac{1}{\delta_{𝒯}}\sum_{i=1}^{I}\frac{\alpha_{𝒯}^{i}x_{0}^{i}Z_{𝒯}^{i}}{\sum_{k=1}^{K}\alpha_{t_{k}}^{i}}\right],\\ 𝒴(0,𝒯) & =-\frac{1}{𝒯}\log E\left[H_{𝒯}\right]=-\frac{1}{𝒯}\log E\left[\frac{1}{\delta_{𝒯}}\sum_{i=1}^{I}\frac{\alpha_{𝒯}^{i}x_{0}^{i}Z_{𝒯}^{i}}{\sum_{k=1}^{K}\alpha_{t_{k}}^{i}}\right]. \end{array}$$ (24)

As (24) indicates, the yield curve shifts in accordance with the total dividend. In detail, as the total dividend $\delta_{𝒯}$ increases, which is equivalent to supply of consumption goods by the market clearing condition, through the expression of the state-price density process, the price of the zero-coupon bond P(0,𝒯) decreases and the yield 𝒴(0,𝒯) increases and vice versa.

### 3.2 Equilibrium between market value processes and wealth processes

Next, we present the equilibrium expression for the market value $S^{j}$ of security *j*.

**Proposition 2.** *The market value* $S^{j}$ *associated with the dividend process* $\delta^{j}$ *in equilibrium is expressed as*


$$\begin{array}{cc} S_{t}^{j} & =\frac{B_{t}}{Z_{t}^{\theta }}\sum_{k:t_{k}\geq t}^{K}E\left[\frac{\delta_{t_{k}}^{j}}{\delta_{t_{k}}}\sum_{i=1}^{I}\frac{x_{0}^{i}\alpha_{t_{k}}^{i}Z_{t_{k}}^{i}}{\sum_{k=1}^{K}\alpha_{t_{k}}^{i}}|{ℱ}_{t}\right]\\ & =\frac{1}{E\left[\frac{1}{\delta_{t_{k}}}\sum_{i=1}^{I}\frac{x_{0}^{i}\alpha_{t_{k}}^{i}}{\sum_{k=1}^{K}\alpha_{t_{k}}^{i}}Z_{t_{k}}^{i}|{ℱ}_{t}\right]}\sum_{k:t_{k}\geq t}^{K}E\left[\frac{\delta_{t_{k}}^{j}}{\delta_{t_{k}}}\sum_{i=1}^{I}\frac{x_{0}^{i}\alpha_{t_{k}}^{i}Z_{t_{k}}^{i}}{\sum_{k=1}^{K}\alpha_{t_{k}}^{i}}|{ℱ}_{t}\right]. \end{array}$$ (25)

**Proof**. See Appendix A.5.

In the following, we show that the total wealth of the *I* agents is equal to the total of the market value of the *N* + 1 securities in equilibrium. We denote the total market value of *N* + 1 securities by $\tilde{S}$ and the aggregate wealth process of agents *I* by *X*, that is,


$$\begin{array}{c} {\tilde{S}}_{t}:=\sum_{j=1}^{N+1}S_{t}^{j}, \end{array}$$

and


$$\begin{array}{c} X_{t}:=\sum_{i=1}^{I}X_{t}^{i}. \end{array}$$

**Proposition 3.** *The total of the market values of N +* 1 *securities in equilibrium* $\tilde{S}$ *has the following expression*


$$\begin{array}{cc} {\tilde{S}}_{t} & =\sum_{j=1}^{N+1}S_{t}^{j}=\frac{B_{t}}{Z_{t}^{\theta }}\sum_{k:t_{k}\geq t}^{K}\sum_{i=1}^{I}\frac{x_{0}^{i}Z_{t}^{i}\alpha_{t_{k}}^{i}}{\sum_{k=1}^{K}\alpha_{t_{k}}^{i}}\\ & =\frac{1}{E\left[\frac{1}{\delta_{k_{t}}}\sum_{i=1}^{I}\frac{x_{0}^{i}\alpha_{k_{t}}^{i}}{\sum_{k=1}^{K}\alpha_{t_{k}}^{i}}Z_{k_{t}}^{i}|{ℱ}_{t}\right]}\sum_{k:t_{k}\geq t}^{K}\sum_{i=1}^{I}\frac{x_{0}^{i}Z_{t}^{i}\alpha_{t_{k}}^{i}}{\sum_{k=1}^{K}\alpha_{t_{k}}^{i}}, \end{array}$$ (26)

*where*


$$\begin{array}{c} k_{t}:=\min\{k\in \{1,\cdots ,K\}:k\geq t\}, \end{array}$$

*In particular, at* $t=k_{t}$,


$$\begin{array}{c} {\tilde{S}}_{t}=S_{k_{t}}=\frac{\delta_{k_{t}}}{\left[\sum_{i=1}^{I}\frac{x_{0}^{i}\alpha_{k_{t}}^{i}}{\sum_{k=1}^{K}\alpha_{t_{k}}^{i}}Z_{k_{t}}^{i}\right]}\sum_{k:t_{k}\geq k_{t}}^{K}\sum_{i=1}^{I}\frac{x_{0}^{i}Z_{k_{t}}^{i}\alpha_{t_{k}}^{i}}{\sum_{k=1}^{K}\alpha_{t_{k}}^{i}}. \end{array}$$

*Moreover,* $\tilde{S}$, *the total of the market values of N +* 1 *securities, equals X, the aggregate wealth process of agents I.*

**Proof**. See Appendix A.6. □

Moreover, the market clearing condition also derives the clearing equations for each security and the money market account as follows.

**Proposition 4.** *Suppose* $\sigma_{S,t}^{j}$*,* j=1,⋯,N+1 *in* (1) *are linearly independent vectors. Then, the market clearing for every risky asset*


$$\begin{array}{c} \sum_{i=1}^{I}\pi_{t}^{i,j,*}=S_{t}^{j},\;j=1,\cdots ,N+1, \end{array}$$

*and the market clearing for the money market account* $\sum_{i}^{I}\pi_{t}^{i,0,*}=0$*, hold.*

**Proof**. See Appendix A.7. □

### 3.3 Remark on recursive structure of volatility processes of the securities

Finally, we remark on the recursive structure in the expression of $S^{j}$ in (25).

The *j*-th market value process $S^{j}$ in (25) is expressed with $Z^{i}$ in (14) that includes $\overset{i}{\underset{t}{\hat{\lambda }}}$, the orthogonal projection of $\lambda_{t}^{i}$ on the space spanned by $\sigma_{S,t}^{j},\;j=1,\ldots ,N+1$. Since $\sigma_{S,t}^{j}$, j=1,…,N+1, represent the volatilities of the security prices $S^{j}$, the pricing expression (25) exhibits a recursive structure, and we must specify ${\hat{\lambda }}^{i}$ in a manner consistent with this recursion when simulating the model.

We note that this recursive structure in $S^{j}$ (25) arises from the incorporation of differences in the agents’ views $\lambda^{i}$ on the fundamental risks represented by Brownian motions, which is associated with some factors that cannot be hedged with these *N* + 1 securities. This incorporation of heterogeneous views provides the model with an incomplete market feature, leading to different state price density processes of agents $H_{t}^{i}=\frac{Z_{t}^{\theta^{i}}}{B_{t}}$ in their individual optimization problems in (11).

In the following, we provide two particular cases to specify $\overset{i}{\underset{t}{\hat{\lambda }}}$ in which the relation (25) holds.

First, when $\delta_{t_{k}}$ is given as a realization of a factor process *y*, that is, $\delta_{t_{k}}=y_{t_{k}},\;k=1,\ldots ,N+1$, assuming that ${\hat{\lambda }}^{i}$ is proportional to its volatility $\sigma_{y}$, we can verify that $\overset{i}{\underset{t}{\hat{\lambda }}}\in Range(\sigma_{S,t}^{⊤})$ as in the following.

**Proposition 5.** *Suppose that* $\delta_{t_{k}},\;k=1,\ldots ,K$ *are given as a realization of a factor process y satisfying*


$$\begin{array}{c} dy_{t}=\mu_{y}y_{t}dt+\sigma_{y}y_{t}dW_{t}. \end{array}$$

*That is,* $\delta_{t_{k}}=y_{t_{k}},\;k=1,\ldots ,K$*. If* $\overset{i}{\underset{t}{\hat{\lambda }}}$ *is proportional to* $\sigma_{y,t}$*, that is,*


$$\begin{array}{c} \overset{i}{\underset{t}{\hat{\lambda }}}=a_{t}^{i}\sigma_{y,t}, \end{array}$$ (27)

*where* $a^{i}$ *is a* ℛ *-valued* $\left\{{ℱ}_{t}\right\}$*-progressively measurable process, then* $\overset{i}{\underset{t}{\hat{\lambda }}}\in Range(\sigma_{S,t}^{⊤})$.

**Proof**. See Appendix A.8.

**Remark 6.** *We note that the volatility processes* $\sigma_{S}^{j}$ *is that of* $S^{j}$ *in* (25)*, which has the expression that includes* $\delta^{j}$ *and* ${\hat{\lambda }}^{i}$*, and, in general, as long as these random variables are specified, the volatility of* $S^{j}$ *is obtained.*

*In this case, with the explicit expression of* $\sigma_{S}^{j}$*, we can easily check whether* $\sigma_{S,t}^{j},\;j=1,\ldots ,N+1$ *are linearly independent, which is required for the clearing condition of the market for securities and the money market account, as we observed in Proposition 4.*

**Remark 7.** *If* ${\hat{\lambda }}^{i}$ *is specified as* $\overset{i}{\underset{t}{\hat{\lambda }}}=a_{t}^{i}\sigma_{y,t}$ *as in* (27)*,* $\sigma_{S,t}^{j}$*,* j=1,…,N+1 *are obtained via* (25)*. Then,* $\lambda_{t}^{i}$ *is decomposed as* $\lambda_{t}^{i}=a_{t}^{i}\sigma_{y,t}⊕\overset{i,⟂}{\underset{t}{\hat{\lambda }}}$*, where* $\overset{i,⟂}{\underset{t}{\hat{\lambda }}}\in Range(\sigma_{S,t}^{⊤}{)}^{⟂}$*.*

Next, when $\delta^{j}$ is the realization of a factor process $y^{j}$, and $\overset{i}{\underset{t}{\hat{\lambda }}}$ is given as a linear combination of $\sigma_{y,t}^{l},\;l=1,\ldots ,N+1$, we have the following expression of $\sigma_{S,t}^{j}$ and confirm $\overset{i}{\underset{t}{\hat{\lambda }}}\in Range(\sigma_{S,t}^{⊤})$.

**Proposition 6.** *Suppose that* $\delta^{j}$ *is the realization of a factor process* $y^{j}$ *that satisfies the following SDEs. That is,* $\delta_{t_{k}}^{j}=y_{t_{k}}^{j}$*,* k=1,…,K*, and*


$$\begin{array}{c} dy_{t}^{j}=\mu_{t}^{j}y_{t}^{j}dt+y_{t}^{j}\sigma_{y,t}^{j}dW_{t}. \end{array}$$

*If* $\overset{i}{\underset{t}{\hat{\lambda }}}$ *is a linear combination of* $\sigma_{y,t}^{l},\;l=1,\ldots ,N+1$*, then* $\sigma_{S,t}^{j}$ *is also a linear combination of* $\sigma_{y,t}^{l},l=1,\ldots ,N+1$ *as follows.*


$$\begin{array}{c} \sigma_{S,t}^{j}=\sigma_{y,t}^{j}+\sum_{i=1}^{I}(\frac{\sum_{t_{k}\geq t}E[\frac{\delta_{t_{k}}^{j}}{\delta_{t_{k}}}A_{t_{k}}^{i}Z_{t_{k}}^{i}|{ℱ}_{t}]}{\sum_{l=1}^{I}\sum_{t_{k}\geq t}E[\frac{\delta_{t_{k}}^{j}}{\delta_{t_{k}}}A_{t_{k}}^{l}Z_{t_{k}}^{l}|{ℱ}_{t}]}-\frac{E[\frac{B_{t_{k}}}{\delta_{t_{k}}}A_{t_{k}}^{i}Z_{t_{k}}^{i}|{ℱ}_{t}]}{E[\frac{B_{t_{k}}}{\delta_{t_{k}}}\sum_{l=1}^{I}A_{t_{k}}^{l}Z_{t_{k}}^{l}|{ℱ}_{t}]})\overset{i}{\underset{t}{\hat{\lambda }}}, \end{array}$$ (28)

*where* $A_{t_{k}}^{i}=\frac{x_{0}^{i}\alpha_{t_{k}}^{i}}{\sum_{k=1}^{K}\alpha_{t_{k}}^{i}}$, i=1,…,I.

*Moreover, if* $\sigma_{S,t}^{j},\;j=1,\ldots ,N+1$ *are linearly independent,* $\overset{i}{\underset{t}{\hat{\lambda }}}\in Range(\sigma_{S,t}^{⊤})$.

**Proof**. See Appendix A.9. □

**Remark 8.** *The expression of* $\sigma_{S,t}^{j}$ *in (28) indicates that* $\sigma_{S,t}^{j},\;j=1,\ldots ,N+1$ *are linear combinations of* $\sigma_{y,t}^{l},\;l=1,\ldots ,N+1$*, which means that the space spanned by* $\sigma_{S,t}^{j},\;j=1,\ldots ,N+1$ *is included in the space spanned by* $\sigma_{y,t}^{l},\;l=1,\ldots ,N+1$*. Since* $\sigma_{S,t}^{j},\;j=1,\ldots ,N+1$ *are linearly independent, both spaces have the same dimension N + 1, indicating that they are the same spaces. Thus, if we denote the space spanned by* $\sigma_{y,t}^{l},\;l=1,\ldots ,N+1$ *by* $Range(\sigma_{y,t}^{⊤})$*, the decomposition of* $\lambda_{t}^{i}$ *is described as follows.*


$$\begin{array}{c} \lambda_{t}^{i}=\overset{i}{\underset{t}{\hat{\lambda }}}⊕\overset{i,⟂}{\underset{t}{\hat{\lambda }}}, \end{array}$$

*where* $\overset{i}{\underset{t}{\hat{\lambda }}}\in Range(\sigma_{y,t}^{⊤})$ *and* $\overset{i,⟂}{\underset{t}{\hat{\lambda }}}\in Range(\sigma_{y,t}^{⊤}{)}^{⟂}$.

## 4 Numerical examples

As an application of the proposed model, this section presents numerical examples based on the equilibrium framework developed in the previous sections. These examples illustrate how the model generates yield curves and how changes in the supply of government bonds influence discount rates and insurance pricing. The Python code used to generate all numerical examples is provided in S1 Code. In particular, we provide equilibrium discount rates for long-term cashflows and the pricing of death benefits and annuities.

### 4.1 Interpretation of the model

In this numerical example, we provide a concrete interpretation of the dividend processes $\delta^{j}$, j=1,…,N+1, and the market value processes $S^{j}$, j=1,…,N+1.

First, the dividend processes, which represent the supply of consumption goods in the model, are interpreted here as the aggregate coupon payments from government bonds and the dividends from stocks. In this numerical setting, the aggregate coupon payments reflect changes in the outstanding notional amounts of government bonds in the secondary market. When the outstanding notional amount increases, the total coupon payments and therefore the dividend processes increase proportionally, and when the outstanding notional amount decreases, the dividend processes decrease. Thus, the dividend processes capture the effect of changes in the outstanding notional amounts of government bonds.

Second, we interpret $S^{j}$ as the market value of security *j*, which is the present value of its future dividends. For j=1,…,N, $S^{j}$ represents the market value of government bonds with time to maturity belonging to zone ${𝒯}_{j}$, and for *j* = *N* + 1, it represents the market value of the stock index.

Changes in the outstanding notional amounts arise from central bank purchases and from government issuance and redemption within each maturity zone. Thus, the process $\delta_{t_{k}}^{j}$ is treated as an exogenous input reflecting these notional changes in zone ${𝒯}_{j}$ caused by the activities of the central bank and the government.

### 4.2 Parameter setting and factor processes

We specify the multi-agent model parameters and the factor processes that drive the dividends and mortality rates used for insurance pricing.

The terminal time is set to *T* = 100 years, and we consider *N* + 1 = 6 securities, consisting of five government bond maturity sectors and a stock price index. Specifically, government bonds are categorized into five maturity sectors: 1–3 years, 3–5 years, 5–10 years, 10–25 years, and beyond 25 years, together with a representative stock price index. We denote these sectors by *j* = 1, 2, 3, 4, 5 and the stock price index by *j* = 6.

We set *I* = 3 agents, each with an initial wealth $x_{0}^{i}=100$, representing three types of institutional investors. For example, the first type may correspond to younger life-cycle investors, the second to older investors, and the third to investors such as banks and insurance companies. Alternatively, these types may be interpreted as domestic private investors, domestic institutional investors, and foreign institutional investors.

We consider 100 discrete time points $t_{k}=k$, k=1,…,100, for dividend and consumption timings. The dimension of the Brownian motion *W* is set to *d* = 8, which exceeds the number of securities *N* + 1 = 6. The drift of the dividend processes depends on an economic factor *Y*^1^, driven by the Brownian motion component *W*_7_.

The mortality rate defined below is influenced by both the economic factor *Y*^1^ and the public health deterioration factor *Y*^2^, driven by *W*_8_. The factors satisfy the following SDEs:


$$\begin{array}{c} dY_{t}^{1}=(lY_{t}^{2}+\mu_{Y,1,t})\;dt+\sigma_{Y,1}\;dW_{7,t},\\ dY_{t}^{2}=\mu_{Y,2,t}\;dt+\sigma_{Y,2}\;dW_{8,t}. \end{array}$$ (29)

We assume that *Y*^1^ and *Y*^2^ are Gaussian processes with positive initial values and positive drifts, representing an economic growth factor and a public health deterioration factor, respectively. The public health deterioration factor negatively affects the drift of *Y*^1^ through *l* < 0. Although Gaussian processes may occasionally take negative values, the parameters $\mu_{Y,1}$, $\mu_{Y,2}$, $\sigma_{Y,1}$, and $\sigma_{Y,2}$ are chosen so that *Y*^1^ and *Y*^2^ remain positive in nearly all simulated sample paths. The mortality rate $\lambda^{m}$ is defined by


$$\begin{array}{c} \lambda_{t}^{m}=(1+k_{1}Y_{t}^{1}+k_{2}Y_{t}^{2})\overset{m}{\underset{t}{\bar{\lambda }}}, \end{array}$$ (30)

so that as the economy grows, the mortality rate decreases, and as disasters occur, the mortality rate increases.

Specifically, for insurance products for individuals of age *t*_0_, we consider the base mortality rate $\overset{m}{\underset{t}{\bar{\lambda }}}$ corresponding to that of a population of age *t*_0_ + *t*. In the following numerical examples, we assume *t*_0_ = 20, covering *t*he mor*t*ality ra*t*e for ages 20–120.

The discrete dividend processes $\delta_{t_{k}}^{j}$ for j=1,…,6 and k=1,…,K are realizations of the following continuous factor processes $y_{t}^{j}$ for j=1,…,6, characterized by the drift and diffusion coefficients $\mu_{y,t}^{j}$ and $\overset{j}{\underset{y}{\bar{\sigma }}}$:


$$\begin{array}{c} dy_{t}^{j}=y_{t}^{j}\left[\mu_{y}^{j}(t,Y_{t})\;dt+\overset{j}{\underset{y}{\bar{\sigma }}}\left(\rho_{j}\;dW_{7,t}+\sqrt{1-\rho_{j}^{2}}\;dW_{j,t}\right)\right], \end{array}$$

where


$$\begin{array}{c} \mu_{y}^{j}(t,Y_{t})=\beta_{Y,j,t}+\alpha_{Y,j,t}Y_{t}^{1}. \end{array}$$ (31)

The parameter $\overset{j}{\underset{y}{\bar{\sigma }}}$ represents the absolute value of the volatility $\sigma_{y}^{j}$ for the factor process $y^{j}$, and $\rho_{j}$ describes the correlation between the Brownian motions driving the economic factor and the *j*-th factor process $y^{j}$. Thus, the dividend processes, corresponding to government bond coupon payments reflecting the outstanding notional amounts of government bonds or dividends from the stock index, have drift components linked to the economic factor and diffusion components correlated with it.

#### 4.2.1 Calibration and parameter choices.

This subsection provides an overview of the parameter choices used in the numerical examples. Although the parameters are not determined by statistical estimation from empirical data, they are selected either to reflect major historical market movements or to fall within ranges commonly used in market practice, and to provide economically meaningful illustrative values for the numerical analysis. The detailed explanations are provided in the following subsections.

Time horizon. Long-term discounting is essential for valuing insurance liabilities and public pension cashflows, which often extend well beyond the 40-year maturity of the longest traded Japanese government bonds (JGBs). Accordingly, we compute yields up to 100 years. This horizon is consistent with life insurance practice for evaluating ultra-long liabilities, which extrapolates the discount curve beyond observable maturities.

The following parameters are chosen to reflect observed mortality rates and major historical market movements.

- **Mortality rates.** The base mortality curve $\overset{m}{\underset{t}{\bar{\lambda }}}$ is constructed from age-dependent mortality statistics published by the Ministry of Health, Labour and Welfare of Japan [15]. The piecewise annual mortality rates (0.04% for ages 20–30, 0.06% for 30–40, 0.10% for 40–50, 0.24% for 50–60, 0.63% for 60–70, 1.7% for 70–80, 4.8% for 80–90, and 40% for 90–100) are based on the mortality rates reported on the cited statistics.
- **Magnitude of supply shocks.** The drift adjustments to $\beta_{Y,3}$, $\beta_{Y,4}$, and $\beta_{Y,5}$ are calibrated to match observed long-term yield movements during major Bank of Japan policy phases. In detail, the 1.4% decline in the 30-year yield during the 2013–2016 QQE period corresponds to a shift of approximately −0.045 in these drift parameters.
- **Expectation shocks.** The adjustment of agents’ subjective views $\left(a^{1},a^{2},a^{3}\right)$ by +0.1 is chosen to match the observed 0.4% increase in the 30-year yield following the Bank of Japan’s exit from yield curve control in March 2024.

The following parameters are not calibrated to empirical data but are chosen at reasonable levels consistent with market practice.

- **Factor dynamics.** The drifts and volatilities of the macro factors $\left(Y_{1},Y_{2}\right)$ are set to $\mu_{Y,1}=\mu_{Y,2}=0.1$ and $\sigma_{Y,1}=\sigma_{Y,2}=0.1$ to ensure *Y*^1^ and *Y*^2^ remain positive for nearly all simulated paths and to represent long-run trends in economic and public health conditions.
- **Dividend factor processes.** The baseline drifts $\beta_{Y,j}$ and volatilities $\overset{j}{\underset{y}{\bar{\sigma }}}$ for the dividend factors $y_{j}$ are selected to reflect relative differences between government bond sectors and the stock index. In particular, $\overset{j}{\underset{y}{\bar{\sigma }}}=0.1,\;j=1,\ldots ,5,$ for bonds and $\overset{6}{\underset{y}{\bar{\sigma }}}=0.3$ for the stock index reflect the higher volatility of equity dividends.
- **Correlation structure.** The correlation parameter $\rho_{j}=0.5,\;j=1,\ldots ,6,$ is chosen as an intermediate value to reflect the correlated dynamics between economic conditions and dividend growth without imposing perfect correlation.
- **Preference heterogeneity.** The time preference parameters $\left(\beta^{1},\beta^{2},\beta^{3})=(0.01,0.02,0.03\right)$ and the subjective risk views $\left(a^{1},a^{2},a^{3})=(1,0,-1\right)$ represent aggressive, neutral, and conservative investor types. These values are selected to illustrate how heterogeneous time preferences and beliefs affect equilibrium discount rates.
- **Initial values.** The initial values $y_{0}^{j}=\frac{\delta_{0}}{10}$ for j=1,…,5 and $y_{0}^{6}=\frac{\delta_{0}}{2}$ are chosen to ensure that the aggregate dividend level is consistent with the consumption-clearing condition and to reflect the scale of outstanding JGBs and prime-sector equities on the Tokyo Stock Exchange.

### 4.3 Base case parameters for factor processes

As base case parameters for the factor processes, we set $Y_{0}^{1}=Y_{0}^{2}=1$, l=−0.003, $\mu_{Y,1}=\mu_{Y,2}=0.1$, $\sigma_{Y,1}=\sigma_{Y,2}=0.1$, $\alpha_{Y,j}=0$ for j=1,…,5, $\alpha_{Y,6}=0.02$, $\beta_{Y,j}=0.01$ for j=1,…,5, $\beta_{Y,6}=0.03$, $y_{0}^{j}=\frac{1}{10}\delta_{0}$ for j=1,…,5, $y_{0}^{6}=\frac{1}{2}\delta_{0}$, $\overset{j}{\underset{y}{\bar{\sigma }}}=0.1$ for j=1,…,5, $\overset{6}{\underset{y}{\bar{\sigma }}}=0.3$, and $\rho_{j}=0.5$ for j=1,…,6, where $\delta_{0}$ is the initial value of the total dividend defined by


$$\begin{array}{c} \delta_{0}=\sum_{i=1}^{I}\frac{x_{0}^{i}}{\sum_{k=1}^{K}\alpha_{t_{k}}^{i}}. \end{array}$$ (32)

We set $\alpha_{Y,j}=0$ for j=1,…,5 because the effect of economic conditions on government bond issuance is not one-directional. In a strong economy, monetary tightening may increase the effective supply of government bonds available in the market, while higher tax revenues may reduce the need for new issuance. Since these effects can offset each other, we set the sensitivity parameter to zero in the base case.

Three agents, denoted by *i* = 1, 2, 3, are considered, each with an initial wealth of $x_{0}^{i}=100$ and time preference parameters $\beta^{1}=0.01$, $\beta^{2}=0.02$, and $\beta^{3}=0.03$. The parameters representing their views on fundamental risks, $a^{i}$ for ${\hat{\lambda }}^{i}$ in (27), are set to *a*^1^ = 1.0, *a*^2^ = 0, and $a^{3}=-1.0$. For example, the first agent has aggressive views with a lower time preference, placing more emphasis on future spending, the second agent has neutral views with moderate time preference, and the third agent has conservative views with a high time preference, prioritizing near-term spending.

Next, by shifting parameters in the model, we examine the impact of changes in bond supply in the market, agents’ expectations about future bond supply, and public health conditions on the long-term discount rate and insurance pricing.

After examining parameter shifts motivated by the historical events discussed below, we compute 100-year discount rates and insurance prices under the shifted parameters.

### 4.4 Supply change impact on the yield curve

First, we examine the impacts of changes in the supply of government bonds on yield curves by shifting the parameters $\beta_{Y,3}$, $\beta_{Y,4}$, and $\beta_{Y,5}$, the constant part of the drift in the dividend processes *y*^3^, *y*^4^, and *y*^5^.

Under the unconventional monetary policy conducted by the former BOJ governor Kuroda, in addition to the negative interest rate policy, large amounts of government bonds were purchased for monetary easing and the effective supply of government bonds available in the market decreased. We observed a 1.4% decrease in the 30-year discount rate from April 2013, when monetary easing began, to July 2016, before the introduction of yield curve control, in which an unlimited amount of bond purchase was attempted to keep the 10-year yield at zero (Ministry of Finance, Japan [16]). This change corresponds to decreasing the parameters $\beta_{Y,3}$, $\beta_{Y,4}$, and $\beta_{Y,5}$ by 0.045.

**Fig 1:**
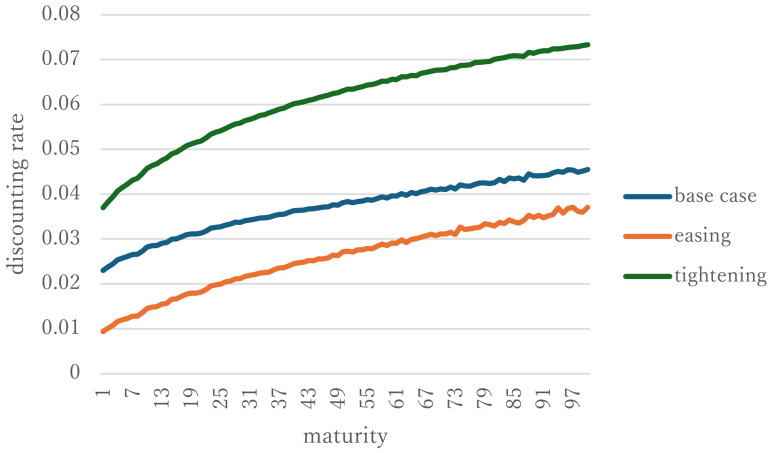
Discount rate for the base, the easing, and the tightening cases. Three cases of yield curves are plotted. $\beta_{Y,3},\beta_{Y,4},\beta_{Y,5}=0.010$ for the base case, $\beta_{Y,3},\beta_{Y,4},\beta_{Y,5}=-0.035$ for the easing case, and $\beta_{Y,3},\beta_{Y,4},\beta_{Y,5}=0.055$ for the tightening case.

As Fig 1 illustrates, the discount rate increases with tightening monetary policy, while it decreases with easing monetary policy. In addition, the yield curves become steeper than in the base case under both easing and tightening. This steepness arises because the parameters with larger values dominate the long-term behavior of the factor processes $y^{j}$ through their exponential form.

In the base case, all values are $\beta_{Y,1},\ldots ,\beta_{Y,6}=0.01$, while in the tightening case $\beta_{Y,3},\beta_{Y,4},\beta_{Y,5}=0.055$ and in the easing case $\beta_{Y,3},\beta_{Y,4},\beta_{Y,5}=-0.035$. As time passes, the total dividend δ becomes increasingly influenced by the factor processes with larger drift parameters, because their exponential growth rates dominate the others. As a result, both the easing and tightening scenarios display steeper yield curves.

### 4.5 Insurance prices

We assume an annual mortality rate $\lambda_{t}^{m}$ in (30) that depends on two factors, namely an unhedgeable economic factor and a public health deterioration factor denoted by *Y*^1^ and *Y*^2^, respectively. Using the yield curve for discounting obtained in the previous subsection, we can price a death benefit that pays $V_{\tau }$ upon death at an exogenously given random time τ, and a life annuity that pays $v_{t}$ annually until time τ.

Following the approach of Chapter 8 in Bielecki and Rutkowski [17], we treat their default time τ as the death time and re-express the second equation in their Proposition 8.2.1 under the physical measure *P*. Their spot martingale measure $Q^{*}$, introduced at the beginning of Section 8.1.1, corresponds in our framework to a measure under which $(S^{j}+D^{j})/B$, j=1,…,N+1, are martingales. For simplicity, we set the density process for the transformation from *P* to $Q^{*}$ equal to $Z^{\theta }$ obtained in the market equilibrium in (21).

Using the associated state price density process $H=Z^{\theta }/B$ in (22), the initial values of the death benefit and the life annuity are given by:

Death benefit value:


$$\begin{array}{c} E\left[V_{\tau }H_{\tau }\right]=E\left[\int_{0}^{\infty }V_{s}\lambda_{s}^{m}\exp\left(-\int_{0}^{s}\lambda_{u}^{m}\;du\right)H_{s}\;ds\right]. \end{array}$$

Here, we set *t* = 0, $\Gamma_{\cdot }=\int_{0}^{\cdot }\lambda_{u}^{m}\;du$, *Z* = *V*, and A=X≡0 in the second equation of Proposition 8.2.1 in [17].

Life annuity value:


$$\begin{array}{c} E\left[\int_{0}^{\infty }v_{s}1_{\left\{s<\tau \right\}}H_{s}\;ds\right]=E\left[\int_{0}^{\infty }v_{s}\exp\left(-\int_{0}^{s}\lambda_{u}^{m}\;du\right)H_{s}\;ds\right], \end{array}$$

where we set *t* = 0, $\Gamma_{\cdot }=\int_{0}^{\cdot }\lambda_{u}^{m}\;du$, $A_{\cdot }=\int_{0}^{\cdot }v_{u}\;du$, and Z=X≡0 in Proposition 8.2.1 of [17].

For simplicity, we set V=v≡1 in both pricing formulas.

We define the base mortality rate ${\bar{\lambda }}^{m}$ as follows: 0.04% for 0≤t<10, 0.06% for 10≤t<20, 0.10% for 20 < *t* < 30, 0.24% for 30≤t<40, 0.63% for 40≤t<50, 1.7% for 50<t≤60, 4.8% for 60<t≤70, 15% for 70<t≤80, 40% for 80<t≤100, which roughly corresponds to the mortality rate for ages 20–100 and above for men in Japan (Ministry of Health, Labour and Welfare [15]). We set $k_{1}=-0.01$ and *k*_2_ = 0.01 in (30), implying that the economic fac*t*or decreases the mortality rate while the public health deterioration factor increases it.

First, in Table 1, the detailed data used for the base, easing, and tightening cases are provided in S1 Data, S2 Data, and S3 Data, respectively. If the drift parameters $\beta_{Y,3}$, $\beta_{Y,4}$, and $\beta_{Y,5}$ of the factor processes *y*^3^, *y*^4^, and *y*^5^, which determine the long-term behavior of the dividend processes, shift from the base value 0.010 to −0.035, corresponding to a reduction in the effective market supply of government bonds due to large-scale central bank purchases, the yields decrease. As a result, the death benefit price increases from 0.093 to 0.165 and the life annuity price increases from 26.5 to 34.7, because a lower discount rate increases the present value of future payments.

**Table 1:** Insurance pricing in the base, easing, and tightening cases.

	Base case	Easing case	Tightening case
Death benefit price	0.093	0.165	0.027
Life annuity price	26.5	34.7	18.1
100-year discount rate	4.6%	3.7%	7.3%

In contrast, if $\beta_{Y,3}$, $\beta_{Y,4}$, and $\beta_{Y,5}$ increase from 0.010 to 0.055, corresponding to an increase in bond supply and therefore higher yields, the death benefit price decreases from 0.093 to 0.027 and the life annuity price decreases from 26.5 to 18.1, because a higher discount rate reduces the present value of future payments.

These insurance price changes are mainly driven by the change in the discount rate. In this scenario, the long-term discount rate for 100 years is 4.6% in the base case, 3.7% under easing, and 7.3% under tightening.

### 4.6 Market expectation change following the Bank of Japan’s announcement of monetary tightening

Finally, we examine how changes in market expectations regarding future bond supply affect long-term discount rates and insurance pricing. We investigate the impact of shifts in market expectations about the outstanding values of assets in the secondary market by adjusting *a*^1^, *a*^2^, and *a*^3^, the parameters representing agents’ views on fundamental risks defined as $\overset{i}{\underset{t}{\hat{\lambda }}}=a_{t}^{i}\sigma_{t}^{\delta }$ in (27).

Following the Bank of Japan’s announcement of its exit from yield curve control in March 2024, market expectations of a future reduction in the BOJ’s government bond purchases intensified. These expectations persisted until the official announcement of reduced bond purchases in July 2024. The heightened expectations led to lower bond prices and higher yields.

Specifically, we observed a 0.4% increase in the 30-year yield between March 2024, when the Bank of Japan exited the yield curve control policy, and July 2024, when the BOJ announced reduced bond purchases (Ministry of Finance, Japan [16]). In our model, an increase in the market outstanding, which is the aggregate value of tradable securities in the secondary market, corresponds to an increase in the dividend process, reflecting higher coupon or stock dividend payments.

This 0.4% increase in the 30-year yield corresponds to shifts of *a*^1^, *a*^2^, and *a*^3^ by 0.1 in (27), representing changes in market expectations regarding the expected return of the secondary market outstanding.

Table 2 shows the impact on insurance pricing via the discount rate resulting from these changes in agents’ views on fundamental risks. The detailed data used for Table 2 are provided in S4 Data.

**Table 2:** Impact of aggressive agents on the 100-year discount rate and insurance prices. *a*^1^ = 1.0, *a*^2^ = 0, $a^{3}=-1.0$ for the base case and *a*^1^ = 1.1, *a*^2^ = 0.1, $a^{3}=-0.9$ for the aggressive case.

	Base case	Aggressive case
Death benefit price	0.093	0.083
Life annuity price	26.5	25.4
100-year discount rate	4.6%	4.8%

If we set *a*^1^ = 1.1, *a*^2^ = 0.1, and $a^{3}=-0.9$, meaning that agents become more aggressive and expect the secondary market outstanding values to increase, the discount rate becomes higher and the death benefit price becomes lower.

### 4.7 Stability analysis and joint shock analysis

#### 4.7.1 Stability analysis.

To examine the robustness of the model, we provide a stability analysis by perturbing several key parameters. Since the main policy implication of the paper is obtained in the easing case ($\beta_{Y,3},\beta_{Y,4},\beta_{Y,5}=-0.035$), we evaluate the sensitivity of this case by slightly modifying the supply parameter around this value. For convenience, let $\beta_{Y,3},\beta_{Y,4},\beta_{Y,5}=\beta$. Specifically, we consider two close values, β=−0.040 and β=−0.030, and compare the resulting long-term yields and insurance prices with those under β=−0.035.

As shown in Table 3, the detailed data used for this stability analysis are provided in S5 Data (β = −0.030) and S6 Data (β = −0.040). Thus, the qualitative implications of the easing case, namely lower long-term yields and higher insurance prices, are maintained under these small perturbations.

**Table 3:** Stability analysis around the easing case.

	β=−0.035	β=−0.030	β=−0.040
Death benefit price	0.165	0.160	0.169
Life annuity price	34.7	34.0	35.4
100-year discount rate	3.71%	3.73%	3.69%

#### 4.7.2 Joint shock analysis.

To further clarify the comparative statics of the model, we also consider scenarios in which the supply shock and the expectation shock occur simultaneously. We examine two economically meaningful joint cases.

First, we consider an easing scenario combined with more aggressive expectations, implemented as β=−0.035 together with $\left(a^{1},a^{2},a^{3})=(1.1,\;0.1,\;-0.9\right)$. Second, we examine a tightening scenario combined with more conservative expectations, implemented as β=0.055 together with $\left(a^{1},a^{2},a^{3})=(0.9,\;-0.1,\;-1.1\right)$.

These combinations correspond to realistic settings in which supply and expectations move together. The detailed data used for Table 4 are provided in S7 Data (tightening–conservative scenario) and S8 Data (easing–aggressive scenario). Table 4 shows that the interaction of supply and expectations produces changes that are consistent with the directions observed in the separate comparative statics analyses in Sections 4.5 and 4.6.

**Table 4:** Base case, easing with aggressive expectations, and tightening with conservative scenarios.

	Base case	Easing + Aggressive	Tightening + Conservative
Death benefit price	0.093	0.144	0.029
Life annuity price	26.5	32.8	18.6
100-year discount rate	4.6%	4.0%	7.2%

## 5 Conclusion

This paper develops a multi-agent equilibrium model in an incomplete market with discrete dividends and heterogeneous beliefs. The model provides a structured setting for examining how supply dynamics and subjective expectations influence long-term discount factors and the valuation of insurance products.

Although the parameters are not selected through statistical estimation from empirical data, they are chosen either to reflect specific historical market movements or to fall within ranges widely used in market practice. The numerical examples illustrate the qualitative implications of the model. In particular, the results show how supply conditions and heterogeneous beliefs affect long-term discount rates in this framework. By varying the drift of the dividend factors and the subjective views of agents, we show how changes in government bond supply or shifts in expectations can influence the long-term discount rate.

The results indicate that long-term discount rates respond to both supply-side changes and changes in investor expectations. The numerical experiments also show how these movements in discount rates can affect the valuation of long-term insurance products such as death benefits and life annuities.

In summary, the contribution of the paper is to provide a tractable equilibrium framework that connects supply dynamics, heterogeneous beliefs, and long-term valuation in an incomplete market. Incorporating statistical estimation to examine these mechanisms with actual data remains a topic for future research.

A Proofs of Theorems and Propositions

A.1 Proof of Proposition 1

First, for fixed $y^{i},\nu^{i}$, we consider for each *k* and sample ω∈Ω,


$$\begin{array}{c} \sup_{c_{k}^{i}\geq 0}[\alpha_{t_{k}}^{i}\eta_{t_{k}}^{i}\log c_{k}^{i}-y^{i}H_{t_{k}}^{i}c_{k}^{i}]. \end{array}$$

This supremum is attained at


$$\begin{array}{c} c_{k}^{i,*}=\frac{\alpha_{t_{k}}^{i}\eta_{t_{k}}^{i}}{y^{i}H_{t_{k}}^{i}}. \end{array}$$

Setting


$$\begin{array}{c} \tilde{U}(y^{i}H_{t_{k}}^{i},t_{k}):=\alpha_{t_{k}}^{i}\eta_{t_{k}}^{i}\log c_{k}^{i,*}-y^{i}H_{t_{k}}^{i}c_{k}^{i,*}, \end{array}$$

we consider


$$\begin{array}{c} \inf_{\nu^{i},\;\sigma_{S}\nu^{i}=0}E\left[\sum_{k=1}^{K}\tilde{U}(y^{i}H_{t_{k}}^{i},t_{k})\right]. \end{array}$$

First, we calculate


$$\begin{array}{c} E\left[\sum_{k=1}^{K}\tilde{U}(y^{i}H_{t_{k}}^{i},t_{k})\right]=E\left[\sum_{k=1}^{K}\alpha_{t_{k}}^{i}\eta_{t_{k}}^{i}\log\left(\frac{\alpha_{t_{k}}^{i}\eta_{t_{k}}^{i}}{y^{i}H_{t_{k}}^{i}}\right)\right]-E\left[\sum_{k=1}^{K}y^{i}H_{t_{k}}^{i}\frac{\alpha_{t_{k}}^{i}\eta_{t_{k}}^{i}}{y^{i}H_{t_{k}}^{i}}\right]\\ =E\left[\sum_{k=1}^{K}\alpha_{t_{k}}^{i}\eta_{t_{k}}^{i}\{\log\alpha_{t_{k}}^{i}+\log\eta_{t_{k}}^{i}-\log y^{i}-\log H_{t_{k}}^{i}\}\right]-\sum_{k=1}^{K}\alpha_{t_{k}}^{i}. \end{array}$$

Thus, we only have to consider the following.


$$\begin{array}{c} \inf_{\nu^{i},\;\sigma_{S}\nu^{i}=0}E\left[-\sum_{k=1}^{K}\eta_{t_{k}}^{i}\log H_{t_{k}}^{i}\right]=\\ \inf_{\nu^{i},\;\sigma_{S}\nu^{i}=0}E[\sum_{k=1}^{K}\eta_{t_{k}}^{i}\{\int_{0}^{t_{k}}r_{s}ds+\frac{1}{2}\int_{0}^{t_{k}}|\theta_{s}+\nu_{s}^{i}{|}^{2}ds+\int_{0}^{t_{k}}(\theta_{s}+\nu_{s}^{i})\cdot dW_{s}\}]. \end{array}$$

We calculate the expectation as follows.


$$\begin{array}{c} E[\sum_{k=1}^{K}\eta_{t_{k}}^{i}\{\int_{0}^{t_{k}}r_{s}ds+\frac{1}{2}\int_{0}^{t_{k}}|\theta_{s}+\nu_{s}^{i}{|}^{2}ds+\int_{0}^{t_{k}}(\theta_{s}+\nu_{s}^{i})\cdot dW_{s}\}]\\ =E^{i}[\sum_{k=1}^{K}\{\int_{0}^{t_{k}}r_{s}ds+\frac{1}{2}\int_{0}^{t_{k}}|\theta_{s}+\nu_{s}^{i}{|}^{2}ds+\int_{0}^{t_{k}}(\theta_{s}+\nu_{s}^{i})\cdot (dW_{s}^{i}+\lambda_{s}^{i}ds)\}]\\ =\sum_{k=1}^{K}E^{i}[\int_{0}^{t_{k}}r_{s}ds+\frac{1}{2}\int_{0}^{t_{k}}(|\theta_{s}{|}^{2}+|\nu_{s}^{i}{|}^{2})ds+\int_{0}^{t_{k}}(\theta_{s}+\nu_{s}^{i})\cdot \lambda_{s}^{i}ds]. \end{array}$$

Here, by (7), $E^{i}[\sum_{k=1}^{K}\int_{0}^{t_{k}}(\theta_{s}+\nu_{s}^{i})dW_{s}^{i}]=0$.

Noting that $\theta \cdot \nu^{i}={\hat{\lambda }}^{i}\cdot \nu^{i}=0$, since


$$\begin{array}{c} \inf_{\nu^{i},\;\sigma_{S}\nu^{i}=0}\sum_{k=1}^{K}E^{i}\left[\int_{0}^{t_{k}}(\frac{1}{2}|\nu_{s}^{i}{|}^{2}+\nu_{s}^{i}\cdot \lambda_{s}^{i})ds\right]=\inf_{\nu^{i},\;\sigma_{S}\nu^{i}=0}\sum_{k=1}^{K}E^{i}\left[\int_{0}^{t_{k}}(\frac{1}{2}|\nu_{s}^{i}+\overset{i,⟂}{\underset{s}{\hat{\lambda }}}{|}^{2})ds\right] \end{array}$$

is attained at $\nu^{i}=-{\hat{\lambda }}^{i,⟂},$ we observe that the infimum is attained at $\nu^{i}=-{\hat{\lambda }}^{i,⟂}.$

Then, we have


$$\begin{array}{c} H_{t}^{i}=\exp[-\int_{0}^{t}r_{s}ds-\frac{1}{2}\int_{0}^{t}|\theta_{s}-\overset{i,⟂}{\underset{s}{\hat{\lambda }}}{|}^{2}ds-\int_{0}^{t}(\theta_{s}-\overset{i,⟂}{\underset{s}{\hat{\lambda }}})\cdot dW_{s}]\\ =\frac{\exp\left[-\frac{1}{2}\int_{0}^{t}|\theta_{s}{|}^{2}ds-\int_{0}^{t}\theta_{s}\cdot dW_{s}\right]}{\exp\left[-\int_{0}^{t}r_{s}ds-\frac{1}{2}\int_{0}^{t}|\overset{i,⟂}{\underset{s}{\hat{\lambda }}}{|}^{2}ds-\int_{0}^{t}\overset{i,⟂}{\underset{s}{\hat{\lambda }}}\cdot dW_{s}\right]}\\ =\frac{Z_{t}^{\theta }}{B_{t}\exp(-\frac{1}{2}\int_{0}^{t}|\overset{i,⟂}{\underset{s}{\hat{\lambda }}}{|}^{2}ds-\int_{0}^{t}\overset{i,⟂}{\underset{s}{\hat{\lambda }}}\cdot dW_{s})} \end{array}$$

where $E^{i}$ denotes the expectation operator under the measure induced by $\lambda^{i}$, i.e., $\frac{dP^{i}}{dP}=\eta_{T}^{i}$, which implies that $dW_{t}=dW_{t}^{i}+\lambda_{t}^{i}dt$, where $W^{i}$ is a Brownian motion under $P^{i}$, by Girsanov’s theorem.

Finally, noting that consumption, dividend and redemption occur at $t_{k}$, k=1,⋯,K with *t*_0_ = 0 and $t_{K}=T$, $y^{i}$ is calculated as


$$\begin{array}{c} \inf_{y^{i}}\sum_{k=1}^{K}E[\eta_{t_{k}}^{i}\alpha_{t_{k}}^{i}\log c_{k}^{i,*}]+y^{i}E\left[x_{0}^{i}-\sum_{k=1}^{K}H_{t_{k}}^{i}c_{k}^{i,*}\right], \end{array}$$ (33)

where


$$\begin{array}{c} c_{k}^{i,*}=\frac{\eta_{t_{k}}^{i}\alpha_{t_{k}}^{i}}{y^{i}H_{t_{k}}^{i}}=\frac{\alpha_{t_{k}}^{i}Z_{t_{k}}^{i}B_{t_{k}}}{y^{i}Z_{t_{k}}^{\theta }}, \end{array}$$ (34)

where we used


$$\begin{array}{c} \frac{\eta_{t_{k}}^{i}}{H_{t_{k}}^{i}}=\frac{Z_{t_{k}}^{i}B_{t_{k}}}{Z_{t_{k}}^{\theta }}. \end{array}$$

Substituting (34) into (33), we observe that this infimum is attained at


$$\begin{array}{c} y^{i}=\frac{\sum_{k=1}^{K}\alpha_{t_{k}}^{i}}{x_{0}^{i}}, \end{array}$$

and thus


$$\begin{array}{c} c_{k}^{i,*}=\frac{x_{0}^{i}}{\sum_{k=1}^{K}\alpha_{t_{k}}^{i}}\frac{\alpha_{t_{k}}^{i}Z_{t_{k}}^{i}B_{t_{k}}}{Z_{t_{k}}^{\theta }}. \end{array}$$

**A.2 Proof of Theorem 1**

We show this by a convex duality argument. Noting that for *y* > 0, $u:{ℛ}^{+}\to ℛ$ is differentiable twice continuously with $u^{\prime }(x)>0,u^{\prime \prime }(x)<0$, and $\tilde{u}(y):=\sup_{x}(u(x)-xy)$,


$$\begin{array}{c} \tilde{u}(y)\geq u(x)-xy\\ \tilde{u}(u^{\prime }(x))=u(x)-xu^{\prime }(x), \end{array}$$

we consider


$$\begin{array}{c} u_{k}(x)=\alpha_{t_{k}}^{i}\eta_{t_{k}}^{i}\log x,\\ u_{k}^{\prime }(x)=\frac{\alpha_{t_{k}}^{i}\eta_{t_{k}}^{i}}{x},\\ u_{k}(x)\leq \sup_{x}(u_{k}(x)-xy)+xy\\ ={\tilde{u}}_{k}(y)+xy \end{array}$$

For $c_{k}^{i,*}=\frac{\eta_{t_{k}}^{i}\alpha_{t_{k}}^{i}}{y^{i}H_{t_{k}}^{i}}$ and arbitrary $c_{k}^{i}$ that satisfy the budget constraint, with $y=y^{i}H_{t_{k}}^{i}$,


$$\begin{array}{c} \eta_{t_{k}}^{i}\alpha_{t_{k}}^{i}\log(c_{k}^{i})\leq {\tilde{u}}_{k}(y^{i}H_{t_{k}}^{i})+c_{k}^{i}y^{i}H_{t_{k}}^{i},\\ {\tilde{u}}_{k}(y^{i}H_{t_{k}}^{i})=\eta_{t_{k}}^{i}\alpha_{t_{k}}^{i}\log(c_{k}^{i,*})-c_{k}^{i,*}y^{i}H_{t_{k}}^{i}, \end{array}$$

where the second equation follows since $u_{k}^{\prime }(c_{k}^{i,*})=y^{i}H_{t_{k}}^{i}$.

By budget constraints, we have the following.


$$\begin{array}{c} \sum_{k=1}^{K}E[c_{k}y^{i}H_{t_{k}}^{i}]\leq x_{0}^{i}, \end{array}$$

Also, since


$$\begin{array}{c} \sum_{k=1}^{K}E[c_{k}^{i,*}y^{i}H_{t_{k}}]=E\left[\eta_{t_{k}}^{i}\alpha_{t_{k}}^{i}\frac{H_{t_{k}}}{H_{t_{k}}^{i}}\right]=y^{i}\sum_{k=1}^{K}\alpha_{t_{k}}^{i}=x_{0}^{i}, \end{array}$$

holds for all state price density processes *H*, $c_{k}^{i,*}$ satisfies the budget constraint for the arbitrary state price density process *H*.

Therefore, we have


$$\begin{array}{c} \sum_{k=1}^{K}E[\eta_{t_{k}}^{i}\alpha_{t_{k}}^{i}\log(c_{k}^{i})]\leq \sum_{k=1}^{K}E[{\tilde{u}}_{k}(y^{i}H_{t_{k}}^{i})]+\sum_{k=1}^{K}E[c_{k}^{i}y^{i}H_{t_{k}}^{i}]\\ \leq \sum_{k=1}^{K}E[{\tilde{u}}_{k}(u_{k}^{\prime }(c_{k}^{i,*}))]+E[c_{k}^{i,*}y^{i}H_{t_{k}}^{i}]=\sum_{k=1}^{K}E[\eta_{t_{k}}^{i}\alpha_{t_{k}}^{i}\log(c_{k}^{i,*})]. \end{array}$$

**A.3 Proof of Theorem 2**

First, we note that if we find $X^{i,*}$ associated with $\left(\pi^{i,*},\pi^{i,0,*}\right)$ such that $X_{t}^{i,*}H_{t}^{i}+\sum_{k:t_{k}<t}c_{k}^{i,*}H_{t_{k}}^{i}$ is a martingale and $X_{T}^{i,*}=0$, $c^{i,*}$ is in the admissible set ${𝒜}^{i}$, since


$$\begin{array}{c} x_{0}=E[\sum_{k:t_{k}<T}c_{k}^{i,*}H_{t_{k}}^{i}]. \end{array}$$

We can find such a wealth process of the agent *i* $X^{i,*}$ by


$$\begin{array}{c} X_{t}^{i,*}=\frac{1}{H_{t}^{i}}E[\sum_{k:t<t_{k}<T}c_{k}^{i,*}H_{t_{k}}^{i}|{ℱ}_{t}] \end{array}$$

since


$$\begin{array}{c} X_{t}^{i,*}H_{t}^{i}+\sum_{k:t_{k}<t}c_{k}^{i,*}H_{t_{k}}^{i}=E[\sum_{k:t_{k}<T}c_{k}^{i,*}H_{t_{k}}^{i}|{ℱ}_{t}]. \end{array}$$

As the optimal consumption of agent *i*’ is $c_{k}^{i,*}=\frac{\eta_{t_{k}}^{i}\alpha_{t_{k}}^{i}}{y^{i}H_{t_{k}}^{i}}$, we calculate


$$\begin{array}{c} \frac{1}{H_{t}^{i}}E[H_{t_{k}}^{i}c_{k}^{i,*}|{ℱ}_{t}]=\frac{1}{H_{t}^{i}}E\left[\frac{\eta_{t_{k}}^{i}\alpha_{t_{k}}^{i}}{y^{i}}|{ℱ}_{t}\right]=\frac{\eta_{t}^{i}}{H_{t}^{i}}\frac{\alpha_{t_{k}}^{i}}{y^{i}}, \end{array}$$

where


$$\begin{array}{c} \eta_{t}^{i}=\exp\left[-\frac{1}{2}\int_{0}^{t}|\lambda_{s}^{i}{|}^{2}ds+\int_{0}^{t}\lambda_{s}^{i}\cdot dW_{s}\right]. \end{array}$$

Then, noting that


$$\begin{array}{c} \frac{\eta_{t}^{i}}{H_{t}^{i}}=\frac{Z_{t}^{i}B_{t}}{Z_{t}^{\theta }}, \end{array}$$

since


$$\begin{array}{c} H_{t}^{i}=\frac{1}{B_{t}}\exp[-\frac{1}{2}\int_{0}^{t}|\theta_{s}-\overset{i,⟂}{\underset{s}{\hat{\lambda }}}{|}^{2}ds-\int_{0}^{t}(\theta_{s}-\overset{i,⟂}{\underset{s}{\hat{\lambda }}})\cdot dW_{s}]\\ =\frac{1}{B_{t}}\exp[-\frac{1}{2}\int_{0}^{t}(|\theta_{s}{|}^{2}+|\overset{i,⟂}{\underset{s}{\hat{\lambda }}}{|}^{2})ds+\int_{0}^{t}(-\theta_{s}+\overset{i,⟂}{\underset{s}{\hat{\lambda }}})\cdot dW_{s}], \end{array}$$

we have


$$\begin{array}{c} \frac{1}{H_{t}^{i}}E[H_{t_{k}}^{i}c_{k}^{i,*}|{ℱ}_{t}]=\frac{\alpha_{t_{k}}^{i}B_{t}Z_{t}^{i}}{y^{i}Z_{t}^{\theta }}. \end{array}$$

Thus, we obtain $X^{i,*}$ as


$$\begin{array}{c} X_{t}^{i,*}=\sum_{k:t_{k}\geq t}^{K}\frac{1}{H_{t}^{i}}E[H_{t_{k}}^{i}c_{k}^{i,*}|{ℱ}_{t}]=\frac{B_{t}Z_{t}^{i}}{y^{i}Z_{t}^{\theta }}\sum_{k:t_{k}\geq t}^{K}\alpha_{t_{k}}^{i},\\ =\frac{B_{t}Z_{t}^{i}}{Z_{t}^{\theta }}\frac{x_{0}^{i}}{\sum_{k=1}^{K}\alpha_{t_{k}}^{i}}\sum_{k:t_{k}\geq t}^{K}\alpha_{t_{k}}^{i}, \end{array}$$ (35)

where we used


$$\begin{array}{c} \frac{1}{y^{i}}=\frac{x_{0}^{i}}{\sum_{k=1}^{K}\alpha_{t_{k}}^{i}}. \end{array}$$

Next, we calculate $\pi^{i,*}$ associated with the wealth process $X^{i,*}$ as follows.

Recalling


$$\begin{array}{c} c_{k}^{i,*}=\frac{B_{t_{k}}Z_{t_{k}}^{i}}{Z_{t_{k}}^{\theta }}\frac{x_{0}^{i}}{\sum_{k=1}^{K}\alpha_{t_{k}}^{i}}\alpha_{t_{k}}^{i}, \end{array}$$

$c_{k}^{i,*}$ is paid from $X^{i,*}$ as consumption in each $t_{k}$.

Accordingly, with $C_{t}^{i,*}:=\sum_{k:t_{k}\leq t}^{K}c_{k}^{i,*}$, and $dC_{t}^{i,*}:=C_{t}^{i,*}-C_{t-}^{i,*}$, applying Ito’s formula to (35), we have


$$\begin{array}{c} dX_{t}^{i,*}=r_{t}X_{t}^{i,*}dt+X_{t}^{i,*}\{\theta_{t}\cdot (\overset{i}{\underset{t}{\hat{\lambda }}}+\theta_{t})dt+(\overset{i}{\underset{t}{\hat{\lambda }}}+\theta_{t})\cdot dW_{t}\}-dC_{t}^{i,*}, \end{array}$$

and for $t\in (t_{k-1},t_{k})$,


$$\begin{array}{c} dX_{t}^{i,*}=r_{t}X_{t}^{i,*}dt+X_{t}^{i,*}\{\theta_{t}\cdot (\overset{i}{\underset{t}{\hat{\lambda }}}+\theta_{t})dt+(\overset{i}{\underset{t}{\hat{\lambda }}}+\theta_{t})\cdot dW_{t}\}. \end{array}$$

The optimal portfolio of agent *i* in equilibrium is calculated as follows.

The optimal portfolio of the agent *i*’ should satisfy


$$\begin{array}{c} (\pi_{t}^{i,*}{)}^{⊤}\sigma_{S,t}=X_{t}^{i,*}(\overset{i}{\underset{t}{\hat{\lambda }}}+\theta_{t}{)}^{⊤}, \end{array}$$

where $\sigma_{S,t}:=$ $\left(\begin{array}{c} \sigma_{S,t}^{1}\\ \vdots \\ \sigma_{S,t}^{N+1} \end{array}\right)$. Then, under the assumption that rank($\sigma_{S,t}$)=*N* + 1 (N+1≤d), that is $\sigma_{S,t}^{1},\cdots ,\sigma_{S,t}^{N+1}$ are linearly independent, *i*’s optimal portfolio $\pi_{t}^{i,*}$ is obtained as


$$\begin{array}{c} \pi_{t}^{i,*}=X_{t}^{i,*}(\sigma_{S,t}\sigma_{S,t}^{⊤}{)}^{-1}\sigma_{S,t}(\overset{i}{\underset{t}{\hat{\lambda }}}+\theta_{t}). \end{array}$$

Finally, the agent *i*’ position in the money market account $\pi^{i,0,*}$ is $X_{t}^{i,*}-\pi_{t}^{i,*⊤}1$.

**A.4 Proof of Theorem 3**

By the market clearing condition (20),


$$\begin{array}{c} \sum_{i=1}^{K}c_{k}^{i,*}=\frac{B_{t_{k}}}{Z_{t_{k}}^{\theta }}\sum_{i=1}^{I}\frac{x_{0}^{i}\alpha_{t_{k}}^{i}Z_{t_{k}}^{i}}{\sum_{k=1}^{K}\alpha_{t_{k}}^{i}}=\delta_{t_{k}}. \end{array}$$

Then, for k=1,…,K we have


$$\begin{array}{c} Z_{t_{k}}^{\theta }=\frac{B_{t_{k}}}{\delta_{t_{k}}}\sum_{i=1}^{I}\frac{x_{0}^{i}\alpha_{t_{k}}^{i}Z_{t_{k}}^{i}}{\sum_{k=1}^{K}\alpha_{t_{k}}^{i}}. \end{array}$$

Since $Z_{t}^{\theta }$ is a martingale, for $t\in (t_{k-1},t_{k}]$,


$$\begin{array}{c} Z_{t}^{\theta }=E\left[\frac{B_{t_{k}}}{\delta_{t_{k}}}\sum_{i=1}^{I}\frac{x_{0}^{i}\alpha_{t_{k}}^{i}Z_{t_{k}}^{i}}{\sum_{k=1}^{K}\alpha_{t_{k}}^{i}}|{ℱ}_{t}\right]. \end{array}$$

For the interest rate *r* in equilibrium, at $t_{k-1}$, noting that


$$\begin{array}{c} \frac{B_{t_{k-1}}}{\delta_{t_{k-1}}}\sum_{i=1}^{I}\frac{x_{0}^{i}\alpha_{k-1}^{i}Z_{t_{k-1}}^{i}}{\sum_{k=1}^{K}\alpha_{k}^{i}}=E\left[\frac{B_{t_{k}}}{\delta_{t_{k}}}\sum_{i=1}^{I}\frac{x_{0}^{i}\alpha_{t_{k}}^{i}Z_{t_{k}}^{i}}{\sum_{k=1}^{K}\alpha_{t_{k}}^{i}}|{ℱ}_{t_{k-1}}\right] \end{array}$$

and $B_{t_{k}}$ is ${ℱ}_{t_{k-1}}$-measurable, since $B_{t_{k}}=B_{t_{k-1}}e^{\int_{t_{k-1}}^{t_{k}}r_{s}ds}$ with *B*_0_ = 1, we have


$$\begin{array}{c} \frac{B_{t_{k}}}{B_{t_{k-1}}}=e^{\int_{t_{k-1}}^{t_{k}}r_{s}ds}=\frac{\frac{1}{\delta_{t_{k-1}}}\sum_{i=1}^{I}\frac{x_{0}^{i}\alpha_{k-1}^{i}Z_{k-1}^{i}}{\sum_{k=1}^{K}\alpha_{k}^{i}}}{E\left[\frac{1}{\delta_{t_{k}}}\sum_{i=1}^{I}\frac{x_{0}^{i}\alpha_{t_{k}}^{i}Z_{t_{k}}^{i}}{\sum_{k=1}^{K}\alpha_{t_{k}}^{i}}|{ℱ}_{t_{k-1}}\right]}, \end{array}$$ (36)

and, equivalently,


$$\begin{array}{c} B_{t_{k}}=B_{t_{k-1}}\frac{\frac{1}{\delta_{t_{k-1}}}\sum_{i=1}^{I}\frac{x_{0}^{i}\alpha_{k-1}^{i}Z_{t_{k-1}}^{i}}{\sum_{k=1}^{K}\alpha_{k}^{i}}}{E\left[\frac{1}{\delta_{t_{k}}}\sum_{i=1}^{I}\frac{x_{0}^{i}\alpha_{t_{k}}^{i}Z_{t_{k}}^{i}}{\sum_{k=1}^{K}\alpha_{t_{k}}^{i}}|{ℱ}_{t_{k-1}}\right]},\;B_{0}=1. \end{array}$$

In particular, $r_{t}$ is obtained as


$$\begin{array}{c} r_{t}=\frac{1}{\left(t_{k}-t_{k-1}\right)}\log\left[\frac{\frac{1}{\delta_{k-1}}\sum_{i=1}^{I}\frac{x_{0}^{i}\alpha_{k-1}^{i}Z_{k-1}^{i}}{\sum_{k=1}^{K}\alpha_{k}^{i}}}{E\left[\frac{1}{\delta_{t_{k}}}\sum_{i=1}^{I}\frac{x_{0}^{i}\alpha_{t_{k}}^{i}Z_{t_{k}}^{i}}{\sum_{k=1}^{K}\alpha_{t_{k}}^{i}}|{ℱ}_{t_{k-1}}\right]}\right] \end{array}$$ (37)

$$\begin{array}{c} B_{t}=B_{t_{k-1}}e^{r_{t}(t-t_{k-1})}\;\text{for }t\in (t_{k-1},t_{k}],\;B_{0}=1. \end{array}$$

**A.5 Proof of Proposition 2**

First, we note that


$$\begin{array}{c} S_{t}^{j}=\frac{B_{t}}{Z_{t}^{\theta }}\sum_{k:t_{k}\geq t}^{K}E\left[\frac{Z_{t_{k}}^{\theta }}{B_{t_{k}}}\delta_{t_{k}}^{j}|{ℱ}_{t}\right]=\frac{B_{t}}{Z_{t}^{\theta }}\sum_{k:t_{k}\geq t}^{K}E\left[\frac{\delta_{t_{k}}^{j}}{\delta_{t_{k}}}\sum_{i=1}^{I}\frac{x_{0}^{i}\alpha_{t_{k}}^{i}Z_{t_{k}}^{i}}{\sum_{k=1}^{K}\alpha_{t_{k}}^{i}}|{ℱ}_{t}\right]. \end{array}$$

In particular, for $t\in (t_{k-1},t_{k}]$, k=1,⋯,K, since


$$\begin{array}{c} Z_{t}^{\theta }=B_{t}E\left[\frac{1}{\delta_{t_{k}}}\sum_{i=1}^{I}\frac{x_{0}^{i}\alpha_{t_{k}}^{i}}{\sum_{k=1}^{K}\alpha_{t_{k}}^{i}}Z_{t_{k}}^{i}|{ℱ}_{t}\right], \end{array}$$

$$\begin{array}{c} \frac{B_{t}}{Z_{t}^{\theta }}=\frac{1}{E\left[\frac{1}{\delta_{t_{k}}}\sum_{i=1}^{I}\frac{x_{0}^{i}\alpha_{t_{k}}^{i}}{\sum_{k=1}^{K}\alpha_{t_{k}}^{i}}Z_{t_{k}}^{i}|{ℱ}_{t}\right]}, \end{array}$$

we have


$$\begin{array}{c} S_{t}^{j}=\frac{1}{E\left[\frac{1}{\delta_{t_{k}}}\sum_{i=1}^{I}\frac{x_{0}^{i}\alpha_{t_{k}}^{i}}{\sum_{k=1}^{K}\alpha_{t_{k}}^{i}}Z_{t_{k}}^{i}|{ℱ}_{t}\right]}\sum_{k:t_{k}\geq t}^{K}E\left[\frac{\delta_{t_{k}}^{j}}{\delta_{t_{k}}}\sum_{i=1}^{I}\frac{x_{0}^{i}\alpha_{t_{k}}^{i}Z_{t_{k}}^{i}}{\sum_{k=1}^{K}\alpha_{t_{k}}^{i}}|{ℱ}_{t}\right]. \end{array}$$

**A.6 Proof of Proposition 3**

First, noting $\sum_{j=1}^{N+1}\delta^{j}=\delta$, the expression of the total of the market values of the securities (26) follows immediately from (25).

Next, by the expression of the optimal wealth (17) for the individual optimization problem, we have


$$\begin{array}{c} X_{t}^{*}:=\sum_{i=1}^{I}X_{t}^{i,*}=\sum_{i=1}^{I}\frac{B_{t}Z_{t}^{i}}{Z_{t}^{\theta }}\frac{x_{0}^{i}}{\sum_{k=1}^{K}\alpha_{t_{k}}^{i}}\sum_{\left\{k:t_{k}\geq t\right\}}^{K}\alpha_{t_{k}}^{i}\\ =\frac{B_{t}}{Z_{t}^{\theta }}\sum_{k:t_{k}\geq t}^{K}\sum_{i=1}^{I}\frac{x_{0}^{i}Z_{t}^{i}\alpha_{t_{k}}^{i}}{\sum_{k=1}^{K}\alpha_{t_{k}}^{i}}. \end{array}$$

Since


$$\begin{array}{c} {\tilde{S}}_{t}=\frac{B_{t}}{Z_{t}^{\theta }}\sum_{k:t_{k}\geq t}^{K}\sum_{i=1}^{I}\frac{x_{0}^{i}Z_{t}^{i}\alpha_{t_{k}}^{i}}{\sum_{k=1}^{K}\alpha_{t_{k}}^{i}}, \end{array}$$

we have $X_{t}={\tilde{S}}_{t}$.

**A.7 Proof of Proposition 4**

For each agent *i*,


$$\begin{array}{c} X_{t}^{i,*}=\pi_{t}^{i,0,*}+\sum_{j=1}^{N+1}\pi_{t}^{i,j,*},\;i=1,\cdots ,I \end{array}$$

Then, a consumption-financed strategy yields that


$$\begin{array}{c} dX_{t}^{i,*}=r_{t}X_{t}^{i,*}dt-dC_{t}^{i,*}+\sum_{j=1}^{N+1}\pi_{t}^{i,j,*}\sigma_{S,t}^{j}dW_{t}^{*},i=1,\cdots ,I, \end{array}$$

where $dW_{t}^{*}=dW_{t}+\theta_{t}dt$, and with $C^{*}:=\sum_{i=1}^{I}C^{i,*}$,


$$\begin{array}{c} dX_{t}^{*}=r_{t}X_{t}^{*}dt-dC_{t}^{*}+\sum_{j=1}^{N+1}\sum_{i=1}^{I}\pi_{t}^{i,j,*}\sigma_{S,t}^{j}dW_{t}^{*}. \end{array}$$

On the other hand, with $\tilde{S}=\sum_{j=1}^{N+1}S^{j}$ and $D:=\sum_{j=1}^{N+1}D^{j}$,


$$\begin{array}{c} d{\tilde{S}}_{t}=r_{t}{\tilde{S}}_{t}dt-dD_{t}+\sum_{j=1}^{N+1}S_{t}^{j}\sigma_{S,t}^{j}dW_{t}^{*}. \end{array}$$

Since $C^{*}=D$ from the consumption market clearing and $X_{t}={\tilde{S}}_{t}$, it must hold that


$$\begin{array}{c} \sum_{j=1}^{N+1}(\sum_{i=1}^{I}\pi_{t}^{i,j,*}-S_{t}^{j})\sigma_{S,t}^{j}=0. \end{array}$$

Then, if $\sigma_{S,t}^{j}$, j=1,⋯,N+1 are linearly independent vectors, we obtain the market clearing for every risky asset,


$$\begin{array}{c} \sum_{i=1}^{I}\pi_{t}^{i,j,*}-S_{t}^{j}=0,\;j=1,\cdots ,N+1, \end{array}$$

and also have the market clearing for the money market account $\sum_{i}^{I}\pi_{t}^{i,0,*}=0$, since


$$\begin{array}{c} {\tilde{S}}_{t}=X_{t}^{*}=\sum_{i=1}^{I}X_{t}^{i,*}=\sum_{j=0}^{N+1}\sum_{i=1}^{I}\pi_{t}^{i,j,*}={\tilde{S}}_{t}+\sum_{i=1}^{I}\pi_{t}^{i,0,*}. \end{array}$$

**A.8 Proof of Proposition 5**

First, noting that


$$\begin{array}{c} d{\tilde{S}}_{t}=\mu_{\tilde{S},t}{\tilde{S}}_{t}dt+{\tilde{S}}_{t}\sigma_{\tilde{S},t}dW_{t}, \end{array}$$

we observe that the Malliavin derivative of ${\tilde{S}}_{t}$ at time *t* is given by


$$\begin{array}{c} D_{t}{\tilde{S}}_{t}={\tilde{S}}_{t}\sigma_{\tilde{S},t}. \end{array}$$

On the other hand, noting that


$$\begin{array}{c} {\tilde{S}}_{t}=\sum_{j=1}^{N+1}S_{t}^{j}=\frac{B_{t}}{Z_{t}^{\theta }}\sum_{i=1}^{I}(\sum_{t_{k}\geq t}A_{t_{k}}^{i})Z_{t}^{i}, \end{array}$$

where $A_{t_{k}}^{i}=\frac{x_{0}^{i}\alpha_{t_{k}}^{i}}{\sum_{k=1}^{K}\alpha_{t_{k}}^{i}}$, we have


$$\begin{array}{c} D_{t}{\tilde{S}}_{t}=D_{t}(\frac{B_{t}}{Z_{t}^{\theta }}\sum_{i=1}^{I}(\sum_{t_{k}\geq t}A_{t_{k}}^{i})Z_{t}^{i})\\ =\frac{B_{t}}{Z_{t}^{\theta }}\sum_{i=1}^{I}(\sum_{t_{k}\geq t}A_{t_{k}}^{i})D_{t}Z_{t}^{i}-\frac{B_{t}}{(Z_{t}^{\theta }{)}^{2}}\sum_{i=1}^{I}(\sum_{t_{k}\geq t}A_{t_{k}}^{i})Z_{t}^{i}D_{t}Z_{t}^{\theta }. \end{array}$$

Using the fact that


$$\begin{array}{c} D_{t}Z_{t}^{i}=\overset{i}{\underset{t}{\hat{\lambda }}}Z_{t}^{i},D_{t}Z_{t}^{\theta }=-\theta_{t}Z_{t}^{\theta }, \end{array}$$

as we observe in the following, we have the following.


$$\begin{array}{c} D_{t}{\tilde{S}}_{t}={\tilde{S}}_{t}\left(\frac{\frac{B_{t}}{Z_{t}^{\theta }}\sum_{i=1}^{I}\left(\sum_{t_{k}\geq t}A_{t_{k}}^{i}\right)\overset{i}{\underset{t}{\hat{\lambda }}}Z_{t}^{i}}{\frac{B_{t}}{Z_{t}^{\theta }}\sum_{i=1}^{I}(\sum_{t_{k}\geq t}A_{t_{k}}^{i})Z_{t}^{i}}+\theta_{t}\right)={\tilde{S}}_{t}\left(\sum_{i=1}^{I}\frac{(\sum_{t_{k}\geq t}A_{t_{k}}^{i})Z_{t}^{i}}{\sum_{l=1}^{I}(\sum_{t_{k}\geq t}A_{t_{k}}^{l})Z_{t}^{l}}\overset{i}{\underset{t}{\hat{\lambda }}}+\theta_{t}\right). \end{array}$$

Hence,


$$\begin{array}{c} \sigma_{\tilde{S},t}=\sum_{i=1}^{I}\frac{(\sum_{t_{k}\geq t}A_{t_{k}}^{i})Z_{t}^{i}}{\sum_{l=1}^{I}(\sum_{t_{k}\geq t}A_{t_{k}}^{l})Z_{t}^{l}}\overset{i}{\underset{t}{\hat{\lambda }}}+\theta_{t}. \end{array}$$ (38)

Next, we investigate the expression of $\theta_{t}$. Since


$$\begin{array}{c} Z_{t}^{\theta }=E[\frac{B_{t_{k}}}{\delta_{t_{k}}}\sum_{i=1}^{I}A_{t_{k}}^{i}Z_{t_{k}}^{i}|{ℱ}_{t}], \end{array}$$

$$\begin{array}{c} D_{t}Z_{t}^{\theta }=-\theta_{t}Z_{t}^{\theta }=E[D_{t}(\frac{B_{t_{k}}}{\delta_{t_{k}}}\sum_{i=1}^{I}A_{t_{k}}^{i}Z_{t_{k}}^{i})|{ℱ}_{t}]\\ =E[\frac{B_{t_{k}}}{\delta_{t_{k}}}\sum_{i=1}^{I}A_{t_{k}}^{i}D_{t}Z_{t_{k}}^{i}-\frac{B_{t_{k}}}{\delta_{t_{k}}^{2}}\sum_{i=1}^{I}A_{t_{k}}^{i}Z_{t_{k}}^{i}D_{t}\delta_{t_{k}}|{ℱ}_{t}]. \end{array}$$

Here, we note that the following relation holds.


$$\begin{array}{c} D_{t}Z_{t_{k}}^{i}=\overset{i}{\underset{t}{\hat{\lambda }}}Z_{t_{k}}^{i}, \end{array}$$

$$\begin{array}{c} D_{t}\delta_{t_{k}}=\sigma_{y,t}\delta_{t_{k}}. \end{array}$$

This follows from $D_{t}\delta_{t_{k}}=D_{t}y_{t_{k}}$, and


$$\begin{array}{c} D_{t}Z_{t_{k}}^{i}=\overset{i}{\underset{t}{\hat{\lambda }}}Z_{t}^{i}+\int_{t}^{t_{k}}\overset{i}{\underset{s}{\hat{\lambda }}}(D_{t}Z_{s}^{i})dW_{s},\\ D_{t}y_{t_{k}}^{i}=\sigma_{y,t}y_{t}+\int_{t}^{t_{k}}\mu_{y,s}(D_{t}y_{s})ds+\int_{t}^{t_{k}}\sigma_{y,s}(D_{t}y_{s})dW_{s}. \end{array}$$

In fact, for fixed *t* and t≤s≤T, $D_{t}y_{s}=\sigma_{y,t}y_{s}$ satisfies


$$\begin{array}{c} d(D_{t}y_{s})=\mu_{y,s}(D_{t}y_{s})+\sigma_{y,s}(D_{t}y_{s})dW_{s},D_{t}y_{t}=\sigma_{y,t}y_{t}. \end{array}$$

and $D_{t}Z_{s}^{i}=\overset{i}{\underset{t}{\hat{\lambda }}}Z_{s}^{i}$ satisfies


$$\begin{array}{c} d(D_{t}Z_{s}^{i})=\overset{i}{\underset{t}{\hat{\lambda }}}(D_{t}Z_{s}^{i})dW_{s},D_{t}Z_{t}^{i}=\overset{i}{\underset{t}{\hat{\lambda }}}Z_{t}^{i}. \end{array}$$

Thus, we obtain


$$\begin{array}{c} -\theta_{t}Z_{t}^{\theta }=E[\frac{B_{t_{k}}}{\delta_{t_{k}}}\sum_{i=1}^{I}A_{t_{k}}^{i}D_{t}Z_{t_{k}}^{i}-\frac{B_{t_{k}}}{\delta_{t_{k}}^{2}}\sum_{i=1}^{I}A_{t_{k}}^{i}Z_{t_{k}}^{i}D_{t}\delta_{t_{k}}|{ℱ}_{t}]\\ =E[\frac{B_{t_{k}}}{\delta_{t_{k}}}\sum_{i=1}^{I}A_{t_{k}}^{i}\overset{i}{\underset{t}{\hat{\lambda }}}Z_{t_{k}}^{i}-\frac{B_{t_{k}}}{\delta_{t_{k}}}\sum_{i=1}^{I}A_{t_{k}}^{i}Z_{t_{k}}^{i}\sigma_{y,t}|{ℱ}_{t}]\\ =Z_{t}^{\theta }(\sum_{i=1}^{I}\frac{E[\frac{B_{t_{k}}}{\delta_{t_{k}}}A_{t_{k}}^{i}Z_{t_{k}}^{i}|{ℱ}_{t}]}{E[\frac{B_{t_{k}}}{\delta_{t_{k}}}\sum_{l=1}^{I}A_{t_{k}}^{l}Z_{t_{k}}^{l}|{ℱ}_{t}]}\overset{i}{\underset{t}{\hat{\lambda }}}-\sigma_{y,t}) \end{array}$$

Hence,


$$\begin{array}{c} -\theta_{t}=\left(\sum_{i=1}^{I}\frac{E[\frac{B_{t_{k}}}{\delta_{t_{k}}}A_{t_{k}}^{i}Z_{t_{k}}^{i}|{ℱ}_{t}]}{E[\frac{B_{t_{k}}}{\delta_{t_{k}}}\sum_{l=1}^{I}A_{t_{k}}^{l}Z_{t_{k}}^{l}|{ℱ}_{t}]}\overset{i}{\underset{t}{\hat{\lambda }}}-\sigma_{y,t}\right), \end{array}$$

and by (38), we have


$$\begin{array}{c} \sigma_{\tilde{S},t}=\sum_{i=1}^{I}\frac{(\sum_{t_{k}\geq t}A_{t_{k}}^{i})Z_{t}^{i}}{\sum_{l=1}^{I}(\sum_{t_{k}\geq t}A_{t_{k}}^{l})Z_{t}^{l}}\overset{i}{\underset{t}{\hat{\lambda }}}-\sum_{i=1}^{I}\frac{E[\frac{B_{t_{k}}}{\delta_{t_{k}}}A_{t_{k}}^{i}Z_{t_{k}}^{i}|{ℱ}_{t}]}{E[\frac{B_{t_{k}}}{\delta_{t_{k}}}\sum_{l=1}^{I}A_{t_{k}}^{l}Z_{t_{k}}^{l}|{ℱ}_{t}]}\overset{i}{\underset{t}{\hat{\lambda }}}+\sigma_{y,t}. \end{array}$$ (39)

Therefore, if $\overset{i}{\underset{t}{\hat{\lambda }}}$ is of the form


$$\begin{array}{c} \overset{i}{\underset{t}{\hat{\lambda }}}=a_{t}^{i}\sigma_{y,t}, \end{array}$$

$\sigma_{\tilde{S},t}$ is proportional to $\sigma_{y,t}$ and thus by (39)


$$\begin{array}{c} \overset{i}{\underset{t}{\hat{\lambda }}}=a_{t}^{i}\sigma_{y,t}\in Range(\sigma_{\tilde{S},t}^{⊤})\subset Range(\sigma_{S,t}^{⊤}). \end{array}$$

**A.9 Proof of Proposition 6**

First, we note that


$$\begin{array}{c} D_{t}\left(S_{t}^{j}\frac{Z_{t}^{\theta }}{B_{t}}\right)=(\sigma_{S,t}^{j}-\theta_{t})\left(S_{t}^{j}\frac{Z_{t}^{\theta }}{B_{t}}\right). \end{array}$$

On the other hand,


$$\begin{array}{c} S_{t}^{j}\frac{Z_{t}^{\theta }}{B_{t}}=\sum_{t_{k}\geq t}E[\frac{\delta_{t_{k}}^{j}}{\delta_{t_{k}}}\sum_{i=1}^{I}A_{t_{k}}^{i}Z_{t_{k}}^{i}|{ℱ}_{t}]. \end{array}$$

Taking the Malliavin derivative $D_{t}$, we observe


$$\begin{array}{c} D_{t}\left(S_{t}^{j}\frac{Z_{t}^{\theta }}{B_{t}}\right)=\sum_{t_{k}\geq t}E[D_{t}(\frac{\delta_{t_{k}}^{j}}{\delta_{t_{k}}}\sum_{i=1}^{I}A_{t_{k}}^{i}Z_{t_{k}}^{i})|{ℱ}_{t}]\\ =S_{t}^{j}\frac{Z_{t}^{\theta }}{B_{t}} \end{array}$$ (40)

Here, for $\delta_{t_{k}}^{j}=y_{t_{k}}^{j}$, since


$$\begin{array}{c} dy_{s}^{j}=\mu_{s}^{j}y_{s}^{j}ds+y_{s}^{j}\sigma_{s}^{y,j}dW_{s}, \end{array}$$

$$\begin{array}{c} D_{t}\delta_{t_{k}}^{j}=\sigma_{y,t}^{j}y_{t_{k}}^{j}. \end{array}$$

Then


$$\begin{array}{c} \sigma_{S,t}^{j}-\theta_{t}=\sigma_{y,t}^{j}-\sigma_{y,t}+\sum_{i=1}^{I}\frac{\sum_{t_{k}\geq t}E[\frac{\delta_{t_{k}}^{j}}{\delta_{t_{k}}}A_{t_{k}}^{i}Z_{t_{k}}^{i}|{ℱ}_{t}]}{\sum_{l=1}^{I}\sum_{t_{k}\geq t}E[\frac{\delta_{t_{k}}^{j}}{\delta_{t_{k}}}A_{t_{k}}^{l}Z_{t_{k}}^{l}|{ℱ}_{t}]}\overset{i}{\underset{t}{\hat{\lambda }}}. \end{array}$$

Therefore, $\sigma_{S,t}^{j}$ is expressed as follows.


$$\begin{array}{c} \sigma_{S,t}^{j}=\theta_{t}+\sigma_{y,t}^{j}-\sigma_{y,t}+\sum_{i=1}^{I}\frac{\sum_{t_{k}\geq t}E[\frac{\delta_{t_{k}}^{j}}{\delta_{t_{k}}}A_{t_{k}}^{i}Z_{t_{k}}^{i}|{ℱ}_{t}]}{\sum_{l=1}^{I}\sum_{t_{k}\geq t}E[\frac{\delta_{t_{k}}^{j}}{\delta_{t_{k}}}A_{t_{k}}^{l}Z_{t_{k}}^{l}|{ℱ}_{t}]}\overset{i}{\underset{t}{\hat{\lambda }}},\\ =-\left(\sum_{i=1}^{I}\frac{E[\frac{B_{t_{k}}}{\delta_{t_{k}}}A_{t_{k}}^{i}Z_{t_{k}}^{i}|{ℱ}_{t}]}{E[\frac{B_{t_{k}}}{\delta_{t_{k}}}\sum_{l=1}^{I}A_{t_{k}}^{l}Z_{t_{k}}^{l}|{ℱ}_{t}]}\overset{i}{\underset{t}{\hat{\lambda }}}-\sigma_{y,t}\right)\\ +\sigma_{y,t}^{j}-\sigma_{y,t}+\sum_{i=1}^{I}\frac{\sum_{t_{k}\geq t}E[\frac{\delta_{t_{k}}^{j}}{\delta_{t_{k}}}A_{t_{k}}^{i}Z_{t_{k}}^{i}|{ℱ}_{t}]}{\sum_{l=1}^{I}\sum_{t_{k}\geq t}E[\frac{\delta_{t_{k}}^{j}}{\delta_{t_{k}}}A_{t_{k}}^{l}Z_{t_{k}}^{l}|{ℱ}_{t}]}\overset{i}{\underset{t}{\hat{\lambda }}}\\ =\sigma_{y,t}^{j}+\sum_{i=1}^{I}\frac{\sum_{t_{k}\geq t}E[\frac{\delta_{t_{k}}^{j}}{\delta_{t_{k}}}A_{t_{k}}^{i}Z_{t_{k}}^{i}|{ℱ}_{t}]}{\sum_{l=1}^{I}\sum_{t_{k}\geq t}E[\frac{\delta_{t_{k}}^{j}}{\delta_{t_{k}}}A_{t_{k}}^{l}Z_{t_{k}}^{l}|{ℱ}_{t}]}\overset{i}{\underset{t}{\hat{\lambda }}}-\sum_{i=1}^{I}\frac{E[\frac{B_{t_{k}}}{\delta_{t_{k}}}A_{t_{k}}^{i}Z_{t_{k}}^{i}|{ℱ}_{t}]}{E[\frac{B_{t_{k}}}{\delta_{t_{k}}}\sum_{l=1}^{I}A_{t_{k}}^{l}Z_{t_{k}}^{l}|{ℱ}_{t}]}\overset{i}{\underset{t}{\hat{\lambda }}}. \end{array}$$

If $\overset{i}{\underset{t}{\hat{\lambda }}}$ is a linear combination of $\sigma_{y,t}^{l},\;l=1,\ldots ,N+1$, each $\sigma_{S,t}^{j},j=1,\ldots ,N+1$ is a linear combination of $\sigma_{y,t}^{l},l=1,\ldots ,N+1$. If $\sigma_{S,t}^{j},j=1,\ldots ,N+1$ in (28) are linearly independent, each $\sigma_{y,t}^{l},\;l=1,\ldots ,N+1$ is expressed as a linear combination of $\sigma_{S,t}^{j},j=1,\ldots ,N+1$, and $\overset{i}{\underset{t}{\hat{\lambda }}}=\sum_{l=1}^{N+1}a_{t}^{l}\sigma_{y,t}^{l}\in Range(\sigma_{S,t}^{⊤})$.

## Supporting information

S1 Code Python code for generating all numerical examples. (PY)

S1 Data Yield curve and insurance pricing data for the base case for Table 1. (CSV)

S2 Data Yield curve and insurance pricing data for the easing case for Table 1. (CSV)

S3 Data Yield curve and insurance pricing data for the tightening case for Table 1. (CSV)

S4 Data Yield curve and insurance pricing data for the aggressive case for Table 2. (CSV)

S5 DataYield curve and insurance pricing data for the case with $\beta_{Y,3}$, $\beta_{Y,4}$, and $\beta_{Y,5}$ set to −0.03 for Table 3.(CSV)

S6 DataYield curve and insurance pricing data for the case with $\beta_{Y,3}$, $\beta_{Y,4}$, and $\beta_{Y,5}$ set to −0.04 for Table 3.(CSV)

S7 Data Yield curve and insurance pricing data for the tightening–conservative scenario for Table 4. (CSV)

S8 Data Yield curve and insurance pricing data for the easing–aggressive scenario for Table 4. (CSV)
